# Cluster-independent marker feature identification from single-cell omics data using SEMITONES

**DOI:** 10.1093/nar/gkac639

**Published:** 2022-07-31

**Authors:** Anna Hendrika Cornelia Vlot, Setareh Maghsudi, Uwe Ohler

**Affiliations:** The Berlin Institute for Medical Systems Biology, Max Delbrück Center for Molecular Medicine, Hannoversche Str. 28, 10115 Berlin, Germany; Department of Computer Science, Faculty of Mathematics and Natural Sciences, Humboldt Universität zu Berlin, Unter den Linden 6, 10099 Berlin, Germany; Department of Computer Science, Faculty of Science, University of Tübingen, 72074 Tübingen, Germany; The Berlin Institute for Medical Systems Biology, Max Delbrück Center for Molecular Medicine, Hannoversche Str. 28, 10115 Berlin, Germany; Department of Computer Science, Faculty of Mathematics and Natural Sciences, Humboldt Universität zu Berlin, Unter den Linden 6, 10099 Berlin, Germany; Department of Biology, Faculty of Life Sciences, Humboldt Universität zu Berlin, Unter den Linden 6, 10099 Berlin, Germany

## Abstract

Identification of cell identity markers is an essential step in single-cell omics data analysis. Current marker identification strategies typically rely on cluster assignments of cells. However, cluster assignment, particularly for developmental data, is nontrivial, potentially arbitrary, and commonly relies on prior knowledge. In response, we present SEMITONES, a principled method for cluster-free marker identification. We showcase and evaluate its application for marker gene and regulatory region identification from single-cell data of the human haematopoietic system. Additionally, we illustrate its application to spatial transcriptomics data and show how SEMITONES can be used for the annotation of cells given known marker genes. Using several simulated and curated data sets, we demonstrate that SEMITONES qualitatively and quantitatively outperforms existing methods for the retrieval of cell identity markers from single-cell omics data.

## INTRODUCTION

Over the last decade, single-cell omics methods have become a commonly used tool across biological domains. Among other modalities, single-cell methods provide a snapshot of the transcriptomic state or genome accessibility state in individual cells using single-cell RNA-sequencing (scRNA-seq) or assays for genome accessibility such as transposase-accessible chromatin (scATAC-seq), respectively. Additionally, spatial transcriptomics methods that capture the gene expression profile at a specific location within a tissue have been gaining popularity in recent years. Taken together, these data types provide a valuable resource for unravelling cell identity, lineage relationships, and the regulation thereof.

Alongside the development of single-cell omics assays, a wealth of specialized tools for the analysis of the resulting data have been developed. Currently, marker features are most often detected by differential testing between computationally inferred clusters of cells. The inherent assumption underlying differential testing-based marker identification is that cells of the same identity are accurately assigned to the same clusters (1). In practice, however, cluster assignment is nontrivial and heavily dependent on pre-processing steps and clustering algorithm parameterization (2). Besides, the concept of distinct cell types is not compatible with systems in which cells lie along a continuous developmental trajectory, like haematopoiesis (3,4) or whole-organism development (5), rendering the clustering of cells into distinct cell types less meaningful. Trajectory or pseudotime inference and analysis provide an alternative to clustering for such systems, but here, too, the uncertainties of cell assignments to branches or pseudotime installments are not commonly taken into account when inferring marker features. Thus, reservations considering group assignments of cells before marker identification persist. In this context, we argue that the definition of a marker feature as a feature that is differentially expressed between clusters of cells is too restrictive. Instead, we propose that a good marker is any feature that characterizes a group of highly similar cells. In other words, marker features are those features that are selectively detected or undetected in a particular cell neighbourhood.

To formalize the notion of a marker feature as a feature that is characteristic for any given group of highly similar cells beyond the concept of cell types or clusters, we developed a method for the cluster-independent identification of marker features from single-cell omics data called SEMITONES (Single-cEll Marker IdentificaTiON by Enrichment Scoring). The method allows for the identification of both local markers, i.e. features that are only detected in a small group of highly similar cells, and global markers, i.e. features that are detected in a larger group of cells covering several cell states. The identification of such markers might help to identify changes in e.g. gene expression or chromatin accessibility that occur during cell identity acquisition. We demonstrate that SEMITONES identifies cell identity markers in published healthy haematopoiesis scRNA-seq and scATAC-seq data (3), spatially resolved gene expression data, and simulated scRNA-seq data, and compare SEMITONES to several popular marker identification methods using published and simulated scRNA-seq data. The results illustrate that SEMITONES accurately and efficiently identifies marker genes and regulatory regions from single-cell omics data.

## MATERIALS AND METHODS

### SEMITONES

**Figure 1.**
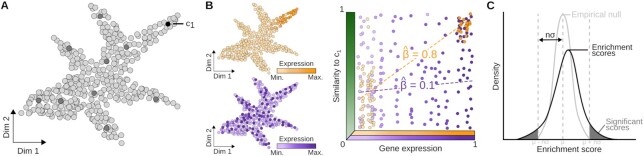
SEMITONES workflow. (**A**) A 2D embedding of all cells where dark grey dots are the selected reference cells and c_1_ is the selected reference cell. (**B**) Linear regression of the similarity to the reference cell (c_1_) and the gene expression quantifies how specifically a gene is expressed in the reference cell neighbourhood. Here, $\hat{{\beta }}$ is proportional to the enrichment score and will be high for neighbourhood-specific genes (orange) and low for neighbourhood-unspecific genes (purple). (**C**) SEMITONES tests for significance by computing a null distribution of enrichment scores from random feature vectors obtained through permutation of the original feature vectors. Significance is declared at *n* standard deviations away from the mean of this empirical null distribution (or the corresponding *P*-value).

In the SEMITONES framework, marker features are defined as features, e.g. genes or open chromatin regions, that are selectively detected in subsets of highly similar cells. To identify these markers, SEMITONES employs a three-step workflow (see Figure 1). In this workflow, SEMITONES first selects a set of cells that are representative of the entire cell population, called reference cells (see Figure 1A). Next, SEMITONES calculates an enrichment score for each feature in each cell using a linear regression framework (see Figure 1B). These enrichment scores are high (positive) for selectively detected features, around zero for uninformative, globally detected or undetected features, and low (negative) for selectively undetected features (see Figure 1B). Finally, SEMITONES performs a statistical test using an empirical null distribution to assess the significance of these enrichment scores (see Figure 1C). The SEMITONES software is freely available from GitHub (https://github.com/ohlerlab/SEMITONES) under the GPL-3.0 license. This repository also includes tutorials for the main SEMITONES functionalities described below.

### Reference cell selection

The default cell selection method in SEMITONES, described in Algorithm 1, aims to select a group of cells that are highly dissimilar to one another (see Figure 1A). To this end, we iteratively select the most dissimilar cell to the previously selected cell. At each iteration, *k* nearest neighbours to the last selected cells are removed from consideration to prevent the algorithm from simply selecting highly dissimilar cells at two extremes of the similarity space. The number of neighbors to be removed from consideration at each iteration (*k*) is determined based on the number of cells in the sample (*N*) and the number of cells to be selected (*n*). The input to the algorithm is cell by feature matrix, *X*, over which cell-to-cell similarities may be computed. For the applications reported in this paper, similarity is computed using a radial basis function (RBF-)kernel over a multidimensional uniform manifold approximation and projection (UMAP) embedding, where the UMAP components are the features that describe each cell. The RBF-kernel has an interpretation as a similarity measure since the kernel value decreases as the Euclidean distance between two vectors increases. The kernel has one free parameter, γ, which can be interpreted as the inverse of the neighbourhood size wherein two vectors may be considered similar (see Supplemental Figure S1a). The algorithm is initialized by creating a set of selected cells *s* and a set of cells to be removed from consideration *e*, each containing the starting cell *i*. Next, the distances of cell *i*, represented by the feature vector *x_i_*, to all cells in the sample (*X*) are computed, the most dissimilar cell is appended to the set *e* and *s*, and the *k*-nearest neighbors to *i* are appended to the set *e*. If several most dissimilar cells exist, one of these cells is selected at random. This process is repeated until *n* cells are selected or, if accounting for rounding-up errors, when there are less than *k* cells left to be selected. Upon convergence, the algorithm outputs a vector of row indices corresponding to the selected cells.

Algorithm 1. SEMITONES reference cell selection.


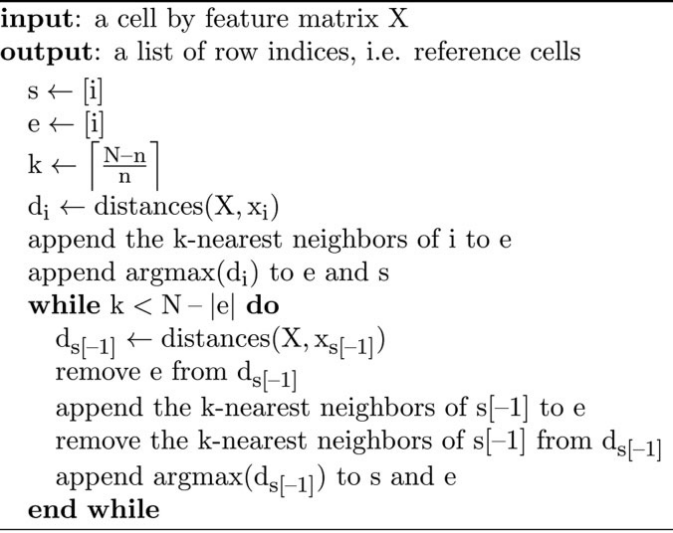


Besides this default algorithm, SEMITONES provides a graphical user interface that can be used to select cells of interest directly from a scatter plot of a 2D cell embedding, e.g. UMAP or t-distributed Stochastic Neighbor Embedding (t-SNE). Additionally, SEMITONES provides a fixed-grid-based selection method by which cells closest to the lattice points of a 2D rectangular grid with a user-defined number of lattice points (see Supplemental Figure S2). In this method, the minimum distance between each pair of selected cells is controlled by a parameter *d* to prevent the selection of disproportionate numbers of cells at the edge of the 2D embedding. This final approach is particularly suitable when 2D cell coordinates are provided, as is the case for spatial transcriptomics data. All three methods are provided in the SEMITONES cell selection module.

### Enrichment scoring

In the context of SEMITONES, a feature is considered a marker if that feature is preferentially detected or undetected in a subset of highly similar cells. From this definition, we derive that marker features harbour a robust linear relationship with the similarity to a reference cell (see Figure 1B). To identify such features, SEMITONES calculates enrichment scores using a simple linear regression framework (Equation 1). Here, y_c_ is a vector containing the similarity to a reference cell *c* using any suitable metric, for which we use an RBF-kernel over the multidimensional UMAP-space, as we did during cell selection. The vector x_f_ represents the value of the feature *f* in each cell. For scRNA-seq or spatial transcriptomics data, this is the gene expression vector. For scATAC-seq, *x*_f_ is an accessibility vector. The regression coefficient $\hat{{\beta }_{c,f}}$, estimated using ordinary least squares, characterizes the strength of the linear relationship between *y*_c_ and *x*_f_. Thus, the value of $\hat{{\beta }_{c,f}}$ is interpreted as a score of the enrichment of some feature *f* in some reference cell *c*. This enrichment score represents how much more likely it is to find high values for *f* at the top of list ranked by their similarity to a cell *c* than at the bottom of said list. Ultimately, features only detected in cells similar to *c* will get a high positive enrichment score, features with non-selective detection scores around 0, and features only detected in cells dissimilar to *c* get low negative scores (Figure 1B).


(1) \begin{equation*} {{y}_c = {x}_f \times \hat{{\beta }_{c,f}} + {\varepsilon }_c,{\varepsilon }_c \, \sim \, N\left( {{\mathrm{0,}}{\sigma }^{\mathrm{2}}} \right)} \end{equation*}

### Significance testing

Although the ranking of genes by their enrichment score in a particular reference cell is the most straightforward way to identify marker genes, SEMITONES also provides a test to identify statistically significantly enriched genes. For this, we shuffle all feature vectors, resulting in randomized feature vectors that resemble the original data. Next, enrichment scores are computed for all randomized feature vectors with respect to each reference cell, resulting in an empirical null distribution for each reference cell (see Figure 1C). Significance can then be declared at a certain number of standard deviations away from the mean of this empirical null distribution, as was done for the analyses presented in this manuscript. Alternatively, significance can be declared based on *P*-values, Bonferroni-corrected *P*-values, and Benjamini–Hochberg FDR-corrected *P*-values provided by SEMITONES.

In addition to lists of significant markers for each reference cell, the empirical null distribution of scores can be used to determine a global marker list. To this end, SEMITONES performs the two-sample Kolmogorov–Smirnov test for goodness of fit between the enrichment scores for each feature in all reference cells and the distribution of the enrichment scores for each permuted feature vector in all reference cells. Genes can then be ranked based on their (corrected) *P*-value.

### Evaluation

#### Identifying marker genes with SEMITONES

The qualitative evaluation of SEMITONES for the identification of marker genes was performed using publicly available scRNA-seq data of healthy human haematopoiesis. The scRNA-seq count matrices were obtained from the GEO database (GSE139369, accessed 28 February 2020) (3). This data set contains 35 582 cells from six samples: two CD34+ enriched BMMC samples, two non-enriched BMMC samples, and two PBMC samples. We removed any cells for which the ratio of the number of genes expressed over the count-depth is greater than or equal to 0.3. Next, we performed scran deconvolution normalization using the computeSumFactors function and clusters obtained from the quickCluster function (6,7). The normalized counts were log-transformed using an alternative pseudo-count proposed by Lun *et al.* (2018) (Lun et al. bioRxiv). A cluster of cells with low count depth in one of the CD34+ cells, identified during a visual inspection of the UMAP embeddings, was removed, leaving 35 156 cells. The (non-normalized) count data of these 35 156 cells were combined, again normalized using scran, and log-transformed using the alternative pseudo-count. We then performed a term frequency-inverse document frequency transformation (tf-idf) on the normalized count data, as in the original publication (3), and reduced the tf-idf transformed data to a 50-dimensional embedding using singular value decomposition. A 2D and 25-dimensional UMAP (n_neighbors = 30 neighbours, min_dist = 0.3) of these 50 dimensions were computed for visualization and similarity calculations, respectively. SEMITONES reference cells were selected using Algorithm 1. Cell similarities for reference cell selection and enrichment scoring were computed using an RBF-kernel with a γ-value of 0.8 over the 25-dimensional UMAP space.

Reference cells were annotated based on the top 10 most highly enriched genes identified by SEMITONES (see Supplemental Table S1). The Blood Atlas (8), with a focus on the Monaco scaled dataset (9), served as a primary reference for cell type-specific marker gene expression. Further marker genes were obtained from the literature (see Supplemental Table S2). Quantitative marker retrieval was evaluated by checking for overrepresentation of highly-ranking genes in the CellMarker database (10). We filtered the human marker database to only contain haematopoietic cells (see Supplemental List 1). We assigned a high-confidence tag to markers that are reported in three or more sources, and a low-confidence tag to markers that were only reported once or twice. Next, we assigned each gene the highest rank it has in any of the reference cells according to SEMITONES. Then, we computed the frequency with which genes in each 25-sized rank bin are present in the CellMarker database subset as high- or low confidence markers.

#### Benchmarking SEMITONES

SEMITONES was qualitatively compared against the alternative marker gene identification methods singleCellHaystack (11) and the default differential expression testing implemented in the Seurat v3 function FindAllMarkers (12). For singleCellHaystack, we use the advanced mode of the highD method using the 25-dimensional UMAP embedding as input. Genes are ranked based on their log(adjusted *P*-value), as returned by singleCellHaystack. Seurat v3 was provided with the biological clusters as provided with the original data publication of the healthy human haematopoiesis scRNA-seq data (3). The only non-default parameter value is return.tresh, which was set to 1 in order to also return non(-significantly) differentially expressed genes. Genes were then ranked by their lowest adjusted *P*-value in any cluster. For comparison to Seurat v3, the SEMITONES gene list was first filtered to only contain those genes that pass the default FindAllMarkers filter. For both the comparison to singleCellHaystack and Seurat v3, genes are ranked by their highest absolute enrichment score in any reference cell. As a result, no distinction is made between presence and absence markers for any of the compared methods.

SEMITONES was also quantitatively compared against several alternative marker identification strategies using synthetic scRNA-seq data simulated using Splatter (13). We simulated 10 cluster-based (using method = ‘groups’ in the splatSimulate function of Splatter) and 10 trajectory-based single-cell datasets (using method = ‘paths’ in the splatSimulate function of Splatter) consisting of 1000 cells in two groups (i.e. clusters or trajectories), 3000 cells in six groups, 5000 cells in 10 groups, 7000 cells in 14 groups, and 10 000 cells in 20 groups. In Splatter, each simulated gene gets assigned a differential expression factor for each group, i.e. cluster or trajectory branch, which serves a ground truth for differential expression. This factor is 1 if a gene is not differentially expressed in a particular group versus all other groups, and greater than or smaller than 1 otherwise. The simulated data was processed using a standard Seurat v3 pipeline to emulate a common initial exploratory analysis of scRNA-seq data. This pipeline consists of normalization (NormalizeData), scaling (ScaleData), dimensionality reduction (RunPCA with npcs = 30, Runumap with max.dim = 10) and clustering (FindClusters) using Seurat v3 (12). Using this simulated data, we compared the performance of SEMITONES against singleCellHaystack (11), Seurat v3’s FindAllMarkers for the Wilcoxon rank-sum test and MAST (12,14), and monocle3’s graph-autocorrelation analysis (15). Again, singleCellHaystack's advanced mode of the highD function was used. For all other methods, default values were used, except for the unfiltered MAST and Wilcoxon rank-sum testing, where min.pct and logfc.threshold were set to 0. SEMITONES was applied using 0.5% of cells as reference cells, selected using Algorithm 1. Performance was assessed based on the area under the receiver operator curve (AUROC) as implemented in scikit-learn v0.24.2 (16). To compute the AUROC, we interpreted 1 – *P*-value as the probability that a gene is differentially expressed for all applied methods.

#### SEMITONES for the identification of cis-regulatory regions

SEMITONES can also be used for identifying neighbourhood-specific (in)accessible chromatin regions from scATAC-seq data. For this, we took scATAC-seq data of healthy human haematopoiesis from the same publication as the scRNA-seq data discussed earlier (3). The scATAC-seq count matrix was downloaded from the GitHub page linked to the original publication (https://github.com/GreenleafLab/MPAL-Single-Cell-2019, accessed on 3 March 2020). This dataset contains 35 038 cells, including CD34+ enriched BMMC, non-enriched BMMC, and PBMC cells. Cells with a peak depth exceeding 200 000 or in which >60 000 peaks were called were removed, leaving 35 022 cells. Peaks were removed if their count exceeded 40 000. The provided cell-by-accessibility matrix was binarized, and we applied a term frequency-inverse document frequency transformation (tf-idf) on the binarized data, as in the original data publication (3), and singular value decomposition to reduce the feature space to 50 dimensions. Next, we computed a 2D and 35-dimensional UMAP (n_neighbours = 50, min_dist = 0.5) over the 50-dimensional space for visualization and similarity calculations, respectively.

Reference cells were selected using Algorithm 1, and cell similarities were computed using an RBF-kernel with a γ-value of 0.8 over the 35-dimensional UMAP space. Reference cells were annotated based on GO-term enrichments and associated genes obtained for all significantly enriched, selectively accessible genes (at >20 standard deviations away from the mean of the empirical null distribution) using GREAT v4.0.4 (17). We used the default association rule and provide all peaks in the dataset as a background set. Motif enrichment for known transcription factor (TF) binding motifs in the significantly enriched, selectively accessible regions was determined using the findMotifsGenome functionality in HOMER v4.10 (18). Motifs were considered enriched if their *q*-value (Benjamini) <0.01. Annotation of nearest genes to peaks, and of peaks as promoters, exons, 5' UTR, 3' UTR, intronic regions, intergenic regions, or transcription termination sites (TTS) was performed using HOMER v4.10. Enhancers were annotated using the permissive enhancer annotations in FANTOM 5 phase 2.6 (19) using the intersect function from bedtools v2.29.2 (20). HOMER annotations were overwritten in favour of enhancer annotations.

#### SEMITONES for the identification of spatially restricted genes

For spatially restricted marker gene identification, SEMITONES was applied to the publicly available Mouse Brain Serial Section 1 (Saggital-Anterior) 10x Genomics Visium spatial transcriptomics data (https://support.10xgenomics.com/spatial-gene-expression/datasets). The data was downloaded using the SeuratData package in R (https://github.com/satijalab/seurat-data). The dataset contains gene expression values for 31 053 genes in 2696 spots whose spatial location in the 2D histological image is known. The gene expression data were normalized using the SCTransform function in Seurat v3 (12) using default parameters, following the spatial dataset vignette provided on the Seurat web page (https://satijalab.org/seurat/articles/spatial_vignette.html), leaving expression data for 17 668 genes in all spots. Reference spots (instead of cells) were selected using the grid-based selection approach with a 6 × 6 grid, while requiring that reference spots are at least 0.2 times the length of the diagonal of a single grid cell apart from one another. Similarities were computed using an RBF-kernel with γ = 0.001 over the 2D coordinates.

## RESULTS

### Identifying marker genes with SEMITONES

**Figure 2.**
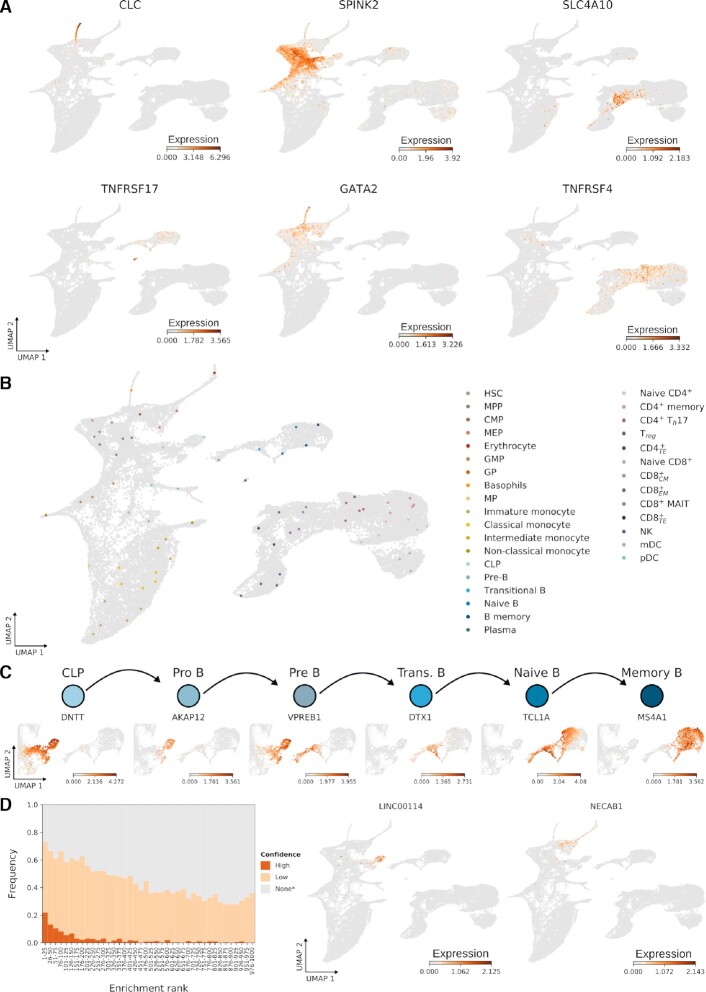
Application of SEMITONES for marker gene identification in scRNA-seq data. Expression profiles and annotations are displayed in 2D UMAP embeddings where each dot represents a cell. (**A**) SEMITONES-retrieved markers of the rare eosinophil/basophil/mast cells and plasma cells (left column), stem-and progenitor cells (middle column), and the CD8 + MAIT cells and Th17 cells subpopulations of T cells (right column). (**B**) Reference cell annotations based on SEMITONES marker genes. (**C**) SEMITONES-retrieved known marker genes of the B cell developmental trajectory. (**D**) The frequency with which SEMITONES markers are present in the CellMarker database (left), and the expression patterns of potential novel marker genes, identified by SEMITONES, that are not in the CellMarker database and have no other literature or database sources supporting their marker gene status in pro B cells and erythrocyte progenitors, respectively (*LINC00114* and *NECAB1*).

We first illustrate that SEMITONES retrieves known marker genes and identifies new marker genes from scRNA-seq data of the well-characterized healthy haematopoietic system (3). The markers retrieved by SEMITONES characterize rare cell types, developmental lineages, and specialized sub-cell types throughout the haematopoietic development. We illustrate top-scoring SEMITONES genes for the rare eosinophil/basophil/mast cell lineage, *CLC* (8), and plasma cells, *TNFRSF17* (21) (Figure 2A). Examples of high-scoring genes that mark more global cell states include *SPINK2*, which marks the HSC and multipotent progenitor (MPP) lineage (22), and *GATA2*, which marks the erythroid-megakaryocyte lineage (4). Finally, SEMITONES-identified markers that highlight specialized T-cell subsets include *SLC4A10* which marks CD8 + mucosal-associated invariation T (MAIT) cells (23) and *TNFRSF4* which marks CD4+ Th17 cells (8).

Using the top-enriched markers for each reference cell, we can annotate all 75 reference cells selected using our data-driven cell selection algorithm (Figure 2B). These annotations broadly correspond to the annotations in the original publication which were obtained using reference protein surface marker expression from the CITE-seq approach and thus serve as a *bona fide* ground truth (see Supplemental Figure S3). Given that we were able to annotate all cell states reported in the original data publication, this result confirms that our data-driven cell selection procedure selected all previously reported cell states. An evaluation of annotations for manually selected reference cells additionally revealed a small population of plasmablasts, which were not identified in the original study (3) nor represented in the algorithmically selected reference cells (see Supplemental Figure S4). Detailed evaluations of the cell type retrieval rate when selecting different proportions of cells, computing distances over different (reduced dimensional) feature spaces, and using different distances metrics, reveal that our approach selects all reported cell types across several parameter spaces (see Supplementary Figure S5a). Additionally, in this regard, SEMITONES’ reference cell selection algorithm outperforms alternative strategies for representative cell subset selection, including random sampling, furthest point sampling, geometric sketching (24), and index cell sampling as implemented in Milo and Wishbone (25,26) (see Supplemental Figure S5).

Besides comprehensive cell-type retrieval, we also visually observe high separation of previously annotated clusters in 2D embeddings obtained using only a few hundred or thousand top-scoring SEMITONES genes, illustrating that SEMITONES identifies subsets of highly biologically informative genes (see Supplemental Figure S6). The separation of cell types in the 2D space when using just 500 and 1000 genes is better when using the top-ranked SEMITONES genes from each reference cell compared to using global top-scoring gene list according to the KS-testing approach (see Methods, see Supplemental Figure S7). Upon further inspection of the top 200 highest scoring genes for the KS-testing approach to genes in the top 10 most enriched genes across reference cells, many genes identified by the KS-testing approach code for transcription factors (TFs) and other lowly expressed genes (see Supplemental List 2). As a result, the KS-testing based ranking of genes across all reference cells highlights biologically relevant genes that might be missed when looking just at the top-ranking genes per reference cell. The observation that SEMITONES identifies informative genes is further supported by comparable or higher density indices for SEMITONES and highly variable genes (HVGs) selected by Seurat v2’s mean-variance-plot (MVP) and Seurat v3’s default HVG selection methods (see Supplemental Figure S7). This density index quantifies how much closer the nearest neighbours are to a given cell relative to the distance between random pairs of cells, and should therefore be maximal (27). In contrast, silhouette scores computed over the top 50 principal components are generally slightly higher when computed over highly variable genes selected by Seurat compared to the same number of top-scoring SEMITONES genes. Here it should be noted that the reference annotations from the original data publication were used to compute the silhouette scores. Since these clusters were obtained by clustering based on the top 3000 most variable features as selected using the MVP from Seurat v.2.3.4 (3), the higher scores might be more related to the greater similarity in data preprocessing than higher consistency within biologically meaningful cell states.

Importantly, in addition to the previously reported cell types, the high granularity of SEMITONES-retrieved markers enabled the annotation of additional monocyte, B- and T-cell subsets based on SEMITONES markers. In Figure 2C, we illustrate markers along the developmental trajectory of B lymphocytes, many of which have well-documented functions and expression patterns. Following the developmental order, the highly enriched *DNTT* gene which codes for the recombination substrate TdT is involved in immunoglobulin and T cell receptor recombination during the lymphoid-primed multipotent progenitors (LMPP) and common lymphoid progenitor (CLP) stages (28,29). Next, the combined enrichment of this *DNTT* gene and *AKAP12* gene allows for the identification of pro-B cells, as *AKAP12* is only expressed in pro-, pre-, and immature B lymphocytes (30). This annotation is further corroborated by the expression profile of the *VPREB1* gene, coding for the ι polypeptide chain of the B-cell receptor (31,32). The top 20 enrichment of *VPREB1* and cell cycle markers like *TOP2A* and *KIFC1* allows for the identification of a subset of highly proliferative large pre-B cells (see Supplemental Table S1) (33). The next developmental state, transitional B lymphocytes, is identified by the top enrichment of the *DTX1* gene which is exclusive to this cell state (34). Finally, naive B lymphocytes are marked by top enrichment of *TCL1A* which is not expressed in memory B cells (35), while memory B cells are marked by the absence of *TCL1A* and the presence of *FCER2* and *MS4A1* among the top 10 enriched genes. Taken together, SEMITONES identifies markers with important biological functions during cell identity acquisition along the full developmental axis.

To validate markers across a broader set of genes, we quantified the enrichment of known markers among the top-scoring SEMITONES genes using the CellMarker database (10) (Figure 2D). For this, we assigned each gene its highest rank in any of the 75 reference cells and divided the top 1000 genes into bins of 25. Then, for each bin, we calculated the frequency with which these genes are high-confidence markers (reported by three or more sources), low-confidence markers (reported by at least one source), or not known marker genes. Here, we observe a clear over-representation of high-and low-confidence marker genes among the top-scoring SEMITONES genes. For the top bin, 73% of genes are present in the CellMarker database, and further literature search revealed evidence of marker gene status for an additional 18% of genes in this bin, increasing the total evidence rate to 91% (see Supplemental Table S3). Genes whose marker status was not previously reported but are in the top 10 ranking genes according to SEMITONES nevertheless show marker-like expression profiles (see Supplemental Figure S8). Examples of such potential novel marker genes include the long non-coding RNA *LINC00114* and the protein-coding *NECAB1* gene for pro-B and erythrocyte progenitors, respectively. As such, we conclude that SEMITONES enables the identification of new relevant genes, even for the well-characterized haematopoietic system.

### Benchmarking SEMITONES for marker gene identification

Next, we compared SEMITONES' marker-retrieval capabilities to those of alternative approaches. We first performed a qualitative comparison of marker identification from the healthy human haematopoiesis scRNA-seq data to the alternative cluster-independent marker identification method singleCellHaystack (11) and the default Wilxocon sum-rank differential testing procedure using the FindAllMarkers function in Seurat v3 (12), which is arguably the most popular marker identification approach for scRNA-seq data. In this comparison, Seurat v3 was provided with the *bona fide* ground-truth biological cluster labels from the original data publication, thus applying the marker identification in a best-case scenario. To compare the gene ranks between the three methods, we ranked genes according to their *P*-value for Seurat v3 and singleCellHaystack and based on their absolute enrichment score for SEMITONES. As such, both presence and absence markers are considered for all approaches.

**Figure 3.**
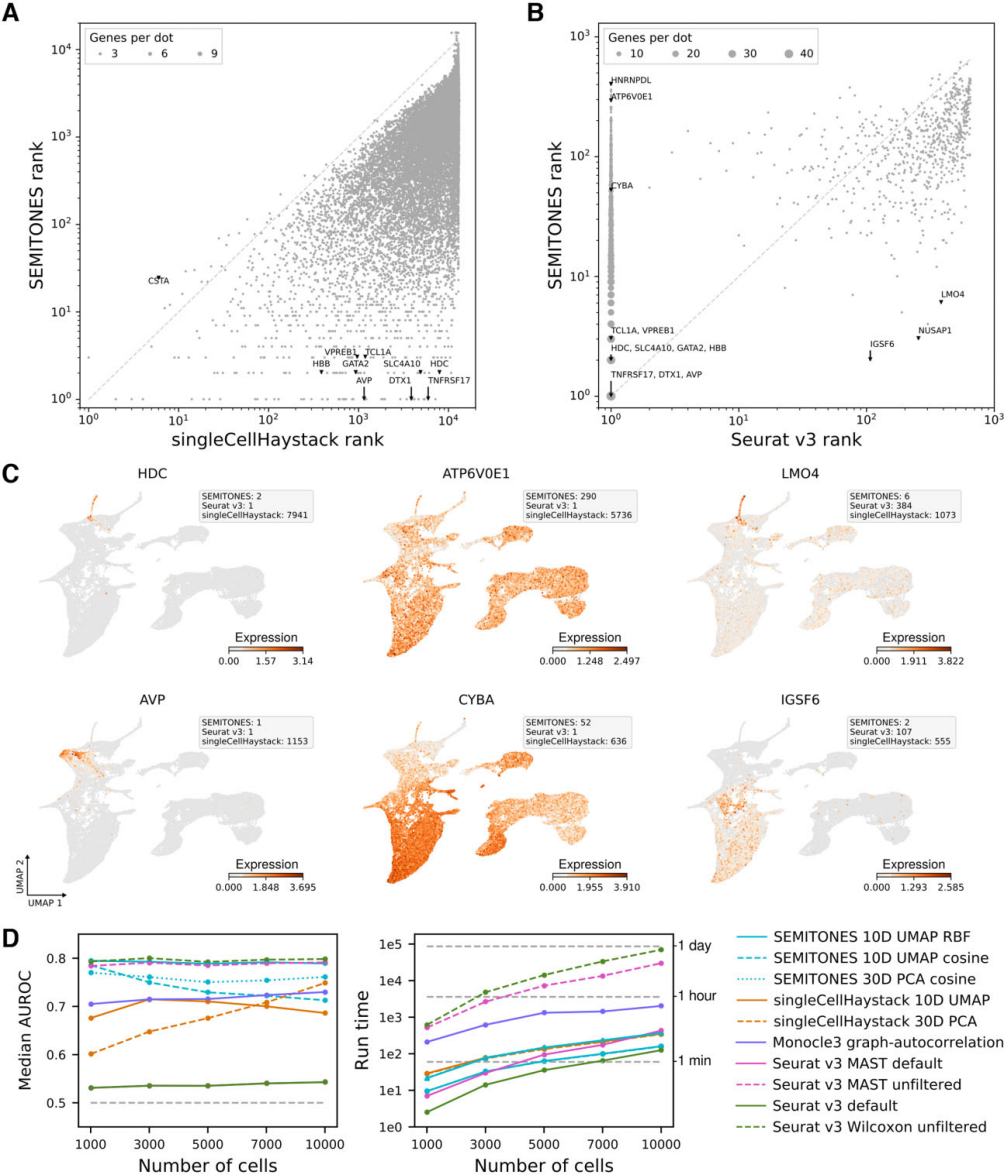
Comparison of SEMITONES to alternative marker identification methods. (**A**) Gene ranks based on SEMITONES enrichment scores differ greatly from gene ranks based on singleCellHaystack adjusted *P*-values. Genes that rank highly according to singleCellHaystack often also do so according to SEMITONES, but several well-known marker genes that ranked highly by SEMITONES are assigned low ranks by singleCellHaystack. (**B**) Several genes ranked lowly by SEMITONES are assigned rank 1, i.e. the *q*-value is 0, by Seurat v3’s default Wilcoxon rank-sum test. Contrastingly, only few high ranking genes according to SEMITONES (rank < 10) rank lowly according to Seurat v3 (rank > 100). (**C**) Example expression profiles of genes that are only identified by SEMITONES and Seurat v3 and not singleCellHaystack (left column), that are only identified by Seurat v3 (middle column), and that are only identified by SEMITONES (right column). (**D**) Comparison of the performance and runtime between SEMITONES and several alternative marker identification methods on simulated scRNA-seq data. Due to runtime constraints, the median AUC of only 12 of the simulated datasets containing 10 000 cells is presented for the unfiltered MAST and Wilcoxon methods. Likewise, the median runtime is given for just 8 and 6 10 000-cell simulations for MAST and Wilcoxon, respectively. In all other cases, the median over all 20 simulations is reported. In the AUROC plot, the grey dashed line indicates the expected AUROC for a random classifier. In the runtime plot, we provide the runtime with (triangle markers) and without (circle markers) significance testing.

We observed limited agreement between SEMITONES and singleCellHaystack (Figure 3A). Many of the markers discussed in the previous section, e.g. *VPREB1*, *HBB*, and *TNFRSF17*, are not among the top-ranking genes for singleCellHaystack. In contrast, the agreement between Seurat v3 and SEMITONES rankings is far higher (Figure 3B). Even so, still roughly 100 genes with a Seurat v3 *P*-value of 0 rank outside the top 100 SEMITONES genes. Similarly, a handful of genes in the top 10 SEMITONES genes falls outside the top 100 for Seurat v3. In Figure 3B, we highlight three types of discrepant ranks, namely genes that are identified as markers by both SEMITONES and Seurat v3 but not singleCellHaystack in the left column, genes that are only identified by Seurat v3 in the middle column, and genes that are only identified by SEMITONES in the right column. Here, we can observe that genes that are not identified by singleCellHaystack are generally genes that are only expressed in a small subset of cells, like the known marker of the eosinophil/basophil/mast cell lineage *HDC* and the HSC marker *AVP*. In contrast, genes that are only identified by Seurat v3 show a differential expression pattern, but are not restricted to a distinct subpopulation of cells. For example, the *CYBA* gene is identified by Seurat v3 as a marker of the HSC cluster with a log-fold change of –0.89, but is not selectively lower expressed in this cluster alone, but instead also in naive T cells, CLPs and the erythrocyte lineage (Figure 3C, see Supplemental Figure S3). Finally, SEMITONES-specific genes include genes that are enriched in small subpopulations of cells but whose expression is not confined to pre-defined cell clusters. An example of this is the *LMO4* gene which shows selective expression in the eosinophil/basophil/mast cell lineage and its progenitors, but not exclusively the eosinophil/basophil/mast cell-lineage cluster as defined in the original study (see Supplemental Figure S3). Similarly, the expression pattern *IGSF6*, which is part of the pre-dendritic cell (pre-DC) signature (4), is highest in just a subset of cells across the granulocyte and monocyte progenitor (GMP), conventional dendritic cell (cDC) and plasmacytoid (pDC) clusters from the original publication. Overall, these results illustrate how the SEMITONES cluster-agnostic marker retrieval approach identifies genes that might be missed by cluster-based approaches, even if *bona fide* ground truth cluster labels are available.

Finally, we quantitatively compared the performance of SEMITONES to singleCellHaystack (11), Seurat v3’s default Wilcoxon rank-sum testing (12), MAST differential expression testing as implemented in Seurat v3’s FindAllMarkers (14), and graph-autocorrelation in monocle3 (15) using simulated scRNA-seq data, for which a ground truth on differential expression is available (see Methods) (13). In terms of the median AUROC score, SEMITONES outperforms alternative clustering-independent methods across most combinations of simulations, multidimensional embeddings, and similarity metrics (Figure 3D, left). Additionally, when using the suggested RBF-kernel over a multidimensional UMAP-embedding, SEMITONES shows comparable performance to differential expression testing using MAST or Wilcoxon rank-sum testing without prior gene filtering. Of note, these differential expression testing methods were provided with the ground-truth clusters and trajectories between which differential expression was simulated, giving them an advantage over alternative methods due to prior information accessibility and using an identical marker gene definition. Importantly, SEMITONES also achieves this performance in a shorter run time of a maximum of 30 minutes for 10000 cells compared to almost an hour for monocle3’s graph-autocorrelation or almost a day for Wilcoxon rank-sum testing or MAST in Seurat v3 on a Linux operating system with a single 3.20GHz core available (Figure 3D, right).

### Neighbourhood-specific cis-regulatory element identification

**Figure 4.**
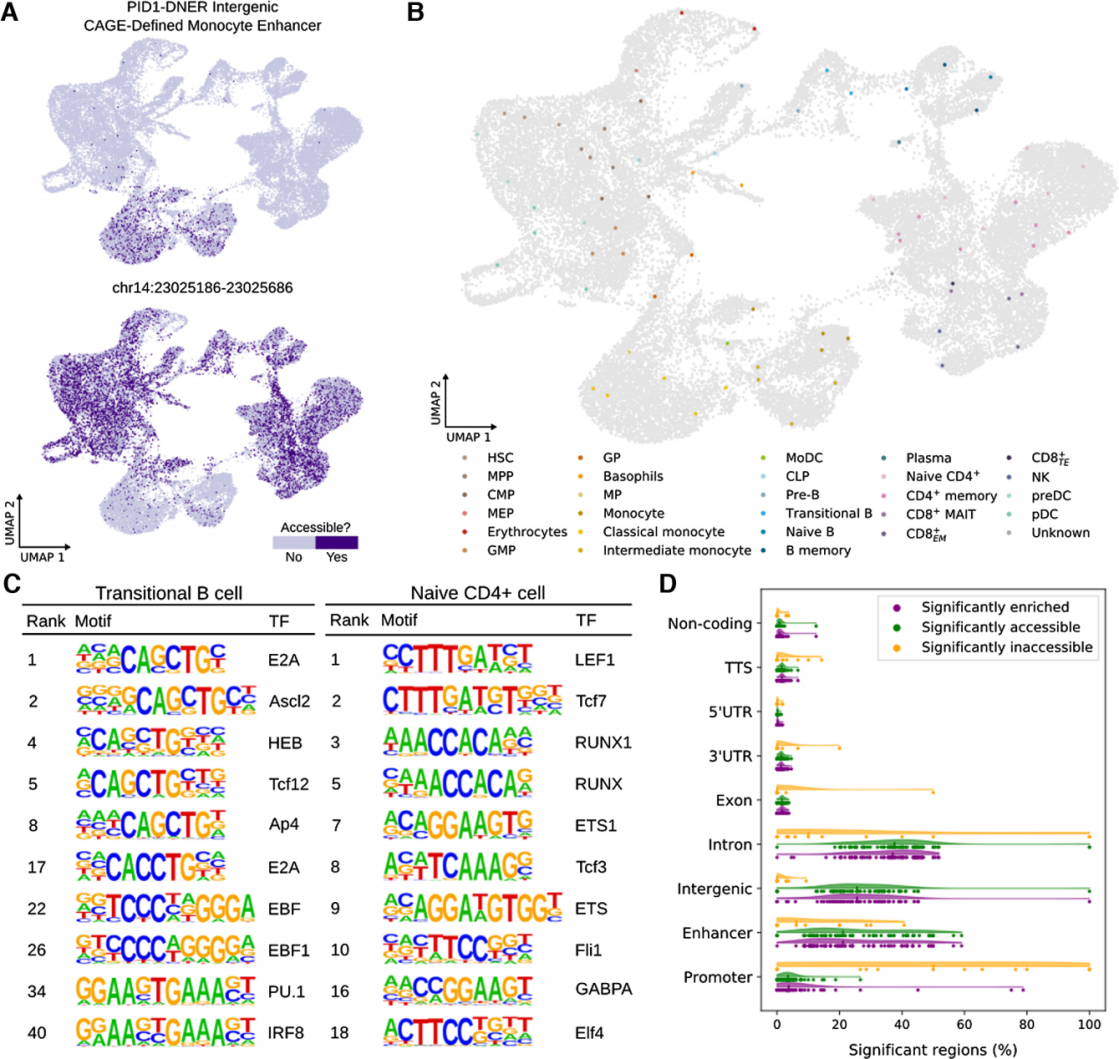
SEMITONES identifies informative peaks from scATAC-seq data. (**A**) Example accessibility profile of a selectively accessible (top) and a selectively inaccessible (bottom) genomic region. (**B**) Reference cell annotations based on GREAT GO-term enrichments and associated genes of significantly enriched, selectively accessible marker peaks identified by SEMITONES. (**C**) Enriched motifs of genomic regions that SEMITONES identified as significantly enriched and selectively accessible in a transitional B reference cell neighbourhood (left) and a naive CD4+ reference-cell neighbourhood (right). (**D**) The distribution of the percentages of all, significantly enriched and selectively accessible, and significantly enriched and selectively inaccessible regions with particular HOMER annotations, or an enhancer annotation in FANTOM5.

Due to the flexibility of its enrichment scoring, SEMITONES is also readily compatible with scATAC-seq count matrices. To showcase this application we use the healthy human scATAC-seq data corresponding to the scRNA-seq data from Granja *et al.* (3). Like for the scRNA-seq data, we use the data-driven selection algorithm to select 75 reference cells and apply SEMITONES to compute enrichment scores for all features, i.e. peaks, in the dataset. Visual inspection of top-scoring peaks confirms that SEMITONES identifies neighbourhood-specific accessible and inaccessible peaks (Figure 4A). Rarely, these regions are known cell-type specific enhancers, like the PID1-DNER intergenic CAGE-defined monocyte enhancer (chr:230147763–230148263). However, cell-type specific annotations of peaks are not commonly available. Thus, to annotate reference cells, we pass significantly selectively accessible peaks to the GREAT algorithm (17) and annotate cells based on associated genes and enriched GO-terms (Figure 4B). In this manner, we annotated 74 out of 75 reference cells. Most annotations correspond to those in the original study, which were obtained using a canonical correlation-based comparison with the corresponding scRNA-seq data (3), with the exception of a few T lymphocyte subpopulations (see Supplemental Figure S9a). As for the scRNA-seq data, dimensionality reduction on only the significantly enriched features improves the separation of clusters in a 2D embedding of the cells (see Supplemental Figure S9b). In terms of quantitative assessments of the informativeness of top-scoring regions, Silhouette scores are higher when using the 5000 to 100 000 most enriched genes according to SEMITONES compared to using the same number of most accessible peaks for dimensionality reduction (see Supplemental Figure S10a). Furthermore, when using the top 500 regions according to SEMITONES, the density indices (27) are substantially higher than when using the top 500 most accessible regions for dimensionality reduction (see Supplemental Figure S10b). When selecting more regions for dimensionality reduction, the density indices for SEMITONES-selected genes drop and are somewhat lower than those for the top accessible regions, although the difference becomes smaller as more regions are selected. These results indicate that SEMITONES is particularly suitable for the selection of a small number of regions that are highly informative of cell identity. Finally, on average 38% of the genes nearest to the significantly enriched elements per reference cell are present as markers in the CellMarker database (10), and on average 17% when only considering high-confidence makers (see Supplemental Figure S10c). Besides recapitulating cell states identified in the original publication, SEMITONES markers revealed a Notch-signalling signature indicative of a pre-T lymphocyte fate in one of the CLPs and a dendritic cell signature in a subset of progenitor cells indicative of a pre-dendritic cell fate. This observation is concordant with the notion that *cis*-regulatory signatures reveal lineage commitments before these are observed on the RNA level (36), suggesting that enhancer accessibility may be more indicative of cell state than gene expression. Besides, these observations reinforce the notion that independent inference on the chromatin level is essential to gain novel insights from scATAC-seq data.

To investigate the biological relevance of the selectively accessible peaks identified by SEMITONES, we determined enriched sequence motifs using HOMER (18), pinpointing binding sites of known lineage-specific TFs, like HOX motifs in stem- and progenitor cells, GATA motifs in the myeloid lineage, and PRDM1 and IRF4 motifs in the B-cell lineage (see Supplemental Table S4). Additionally, known regulators of B cell differentiation, like E2A, EBF, PU.1 and IRF8 (37), are enriched in pre-B and transitional B cells (Figure 4C, see Supplemental Table S4). Likewise, many of the motif enrichments in naive CD4 + cells have known functions in CD4 + cell specialization (38–41). Strikingly, many of the transcription factors identified by our KS-test based gene ranking approach (see Supplemental List 2) correspond to the transcription factors whose binding motifs were found to be enriched in selectively accessible peaks (see Supplemental Table S4). Taken together, these results corroborate that SEMITONES identifies functionally relevant, cell subset-specific accessible regions from scATAC-seq data.

SEMITONES also identifies selectively inaccessible regions. Figure 4D shows the proportion of all significantly enriched, significantly enriched and accessible, and significantly enriched and inaccessible peaks that have a certain annotation across reference cells. Selectively inaccessible peaks are most likely to overlap promoter regions, while selectively accessible regions are more likely to be enhancers (also when correcting for the total number of peaks with a given annotation, see Supplemental Figure S11). These trends fit prior observations that promoters default to accessibility across conditions, while distal regulatory elements exhibit condition-specific accessibility profiles (42).

### SEMITONES identifies spatially restricted genes

**Figure 5.**
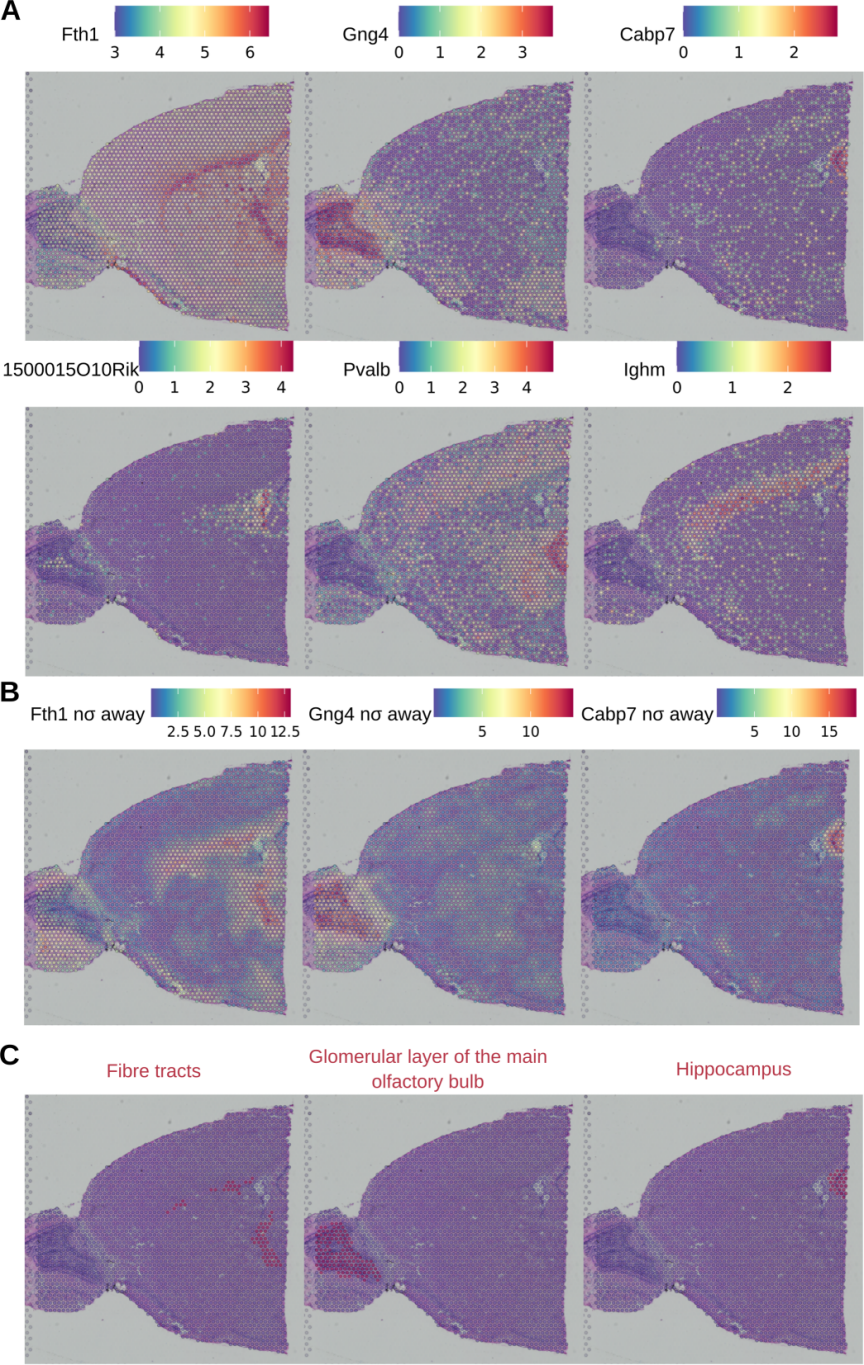
SEMITONES identifies spatially restricted markers from spatial transcriptomics data. (**A**) The spatially resolved expression profile of top spatially enriched SEMITONES genes. (**B**) The significance level in the number of standard deviations away from the mean of the empirical null distribution for a marker gene of fibre tracts (*Fth1*, left), a marker of the glomerular layer of the main olfactory bulb (*Gng4*, middle), and a marker of the hippocampus (*Cabp7*, right). (**C**) The annotation of spots as belonging to the fibre tracts (left), the glomerular layer of the main olfactory bulb (middle), and the hippocampus (right) based on the enrichment scores of known marker genes for these regions (*Fth1*, *Gng4*, *Capb7*). Cells are annotated as belonging to a certain spatial structure if their enrichment score is at least 8 standard deviations higher than the mean of the emprical null distribution.

Given the recent increase in the availability of spatial transcriptomics datasets, we demonstrate how SEMITONES can be applied to gene expression data with known 2D coordinates of each transcriptional profile. For this, we use a publicly available 10x Visium spatial transcriptomics dataset of the anterior mouse brain. Since the 2D coordinates of each profile are given, reference cells are selected using a grid-based reference cell selection method (see Methods). SEMITONES can then be used to identify marker genes for specific brain structures like the fibre tracts (*Fth1*), the glomerular layer of the main olfactory bulb (*Gng4*), the hippocampus (*Cabp7*), the choroid plexus (*1500015O10Rik*), the reticular thalamus (*Pvalb*), and cortical layer V (*Ighm*) (43) (Figure 5A).

To compare SEMITONES results to alternative clustering-free methods, we checked how many of the top 100 SEMITONES genes are also among the significant genes called by the variogram method in Seurat v3. 96 out of these 100 genes are among the 1910 significantly spatially variable genes identified by the variogram method. The remaining four genes show low spatially restricted expression in a small subset of cells (see Supplemental Figure S12). These genes include the hypothalamus-enriched *Magel2* and *Peg10* genes (43), indicating that SEMITONES identifies markers even for genes with high noise (i.e. drop-out) levels. Overall, these results illustrate that SEMITONES is a viable method for spatially restricted marker identification.

Finally, SEMITONES can also be used in an inverse manner to annotate cells given a set of known markers. To do this, we computed the enrichment scores for just the given marker genes in all spots (equivalent to cells in scRNA-seq data). To enable a fine-grained final annotation, we increased the value of γ to 0.01 to consider smaller cell neighbourhoods during enrichment scoring. Figure 5B illustrates the enrichment level for the fibre tract marker *Fth1*, the glomerular layer of the main olfactory bulb marker *Gng4*, and the hippocampus marker *Cabp7*. The annotations of the corresponding regions are given at eight standard deviations above the mean of the empirical null distribution in Figure 5C. These annotations overlap largely with the brain structures in the histological image, confirming that SEMITONES enrichment scoring and significance testing allows for the annotation of spatial tissue structures. Although we chose to illustrate this application on spatial transcriptomics data to enable a straightforward visualization of the results, the approach can also be used on scRNA-seq data, as illustrated by the recent application for the annotation of a single-cell *Arabidopsis*-root expression atlas (44).

## DISCUSSION

We present SEMITONES, a tool for the *de novo*, cluster-independent identification of informative features from single-cell omics data. By omitting cell clustering, we aim to mitigate error and bias propagation from cell identity assignments. Using scRNA-seq, scATAC-seq and spatial transcriptomics data, we illustrate how SEMITONES identifies biologically relevant features, i.e. genes or accessibility peaks, from diverse single-cell omics data types. For both scRNA-seq and scATAC-seq, the selected features contain enough biologically relevant information to annotate reference cells and to improve the cell state separation in lower-dimensional embeddings compared to using all features. This observation may be of particular interest in the context of targeted sequencing approaches, which use a subset of highly informative genes to characterize a specific system. Additionally, we use simulated scRNA-seq data to verify the accuracy of SEMITONES, to showcase that SEMITONES achieves comparable performance to differential testing even when differential testing methods are provided with ground truth cluster labels, and establish its superior performance over alternative clustering-independent feature identification methods for single-cell transcriptomic data. Although simulated scATAC-seq data with a ground-truth on differential accessibility is not currently available and no high-quality reference databases for cell type-specific accessible chromatin regions exist, the enriched sequence motifs and their correspondence to transcription factors that were identified as markers from scRNA-seq data illustrate that SEMITONES identifies biologically informative peaks. Finally, we illustrate how the inverse application of SEMITONES can also be used for the annotation of individual cells given specific marker genes.

The benchmark on simulated scRNA-seq data also illustrates SEMITONES' dependence on the similarity metric and the embedding over which this similarity metric is computed. First and foremost, using an RBF-kernel over a multidimensional UMAP embedding results in the highest performance for SEMITONES, endorsing its use for other applications presented in this manuscript. Another motivation for the RBF-kernel is the interpretation of its γ parameter as an inverse measure of the neighbourhood size to be considered during enrichment scoring (see Supplemental Figure S1a). By choosing a sufficiently large value for γ, one ensures that local markers, i.e. markers selective for a small subset of cells, are detected. More global markers will also be identified as significantly enriched, but will be assigned enrichment scores that rank lower than these local markers. Conversely, if the γ-value is too low, highly selectively markers will not be selected because cells in a larger neighbourhood will be considered to be highly similar. The same holds true when using alternative metrics where similarities for larger neighbourhoods of cells are very high, such as the cosine similarity (see Supplemental Figure S1b), potentially resulting in lower performance for larger, more complex (simulated) datasets (Figure 3D). The selection of this similarity metric is also relevant in the context of algorithmic reference cell selection (see Supplemental Figure S5). Here, too, using an RBF-kernel over a multidimensional UMAP embedding results in competitive performance.

The interpretation of the RBF-kernel as defining cell-neighbourhoods for which to identify markers, indexed by reference cells, does not suffer from the same limitations as clustering-based approaches. Most importantly, in non-fuzzy clustering approaches often used for differential expression testing, the inclusion of a cell in one cluster imposes the exclusion of that cell from all other clusters. Conversely, in SEMITONES, a cell being highly similar to one reference cell does not exclude the possibility of that same cell being highly similar to another reference cell. As a result, neighbourhoods characterized by the RBF-kernel may be overlapping. In this regard, the neighbourhood definition shows similarities to the overlapping neighbourhoods used in Milo (25), where overlapping cell neighbourhoods are constructed by the assignment of cells to index, i.e. reference, cells on a kNN-graph. Here, a critical difference is that the assignments to the index cell in Milo are hard assignments, while the similarity to the reference cell provides a measure of how similar a cell is to the reference cell, equivalent to a fuzzy neighbourhood assignment. Due to the independence of the reference cell similarities, selecting more reference cells than cell types or states does not impact the descriptiveness of the enrichment scores per reference cell. This independence of the reference cells has several advantages. Firstly, enrichment scoring can be performed independently per reference cell so the enrichment score computations can be run in parallel. However, even so, selecting a very large number of reference cells creates the need for post-processing and merging of results from reference cells with highly similar results, thus it is generally not advisable to select too large a number of reference cells. Fortunately, due to the independence of the reference score computations, additional reference cells may be manually selected *post hoc* in case the user feels that not all desired cell neighbourhoods were explored using automatically selected cells, as illustrated by the identification of a plasmablast neighbourhood by manually adding select reference cells (see Supplemental Figure S4).

The flexibility of SEMITONES is illustrated by its applicability to diverse data types, like the identification of biologically relevant genomic regions from scATAC-seq data and the identification of brain region-specific genes from spatial transcriptomics data. Both of these applications also show SEMITONES' robustness to sparsity and noise. Namely, scATAC-seq is notoriously sparse, with generally <3% of entries being non-zero, both because many chromatin regions are not accessible in any given cell and due to high drop-out rates. Yet, SEMITONES identifies biologically informative peaks from scATAC-seq data. Additionally, SEMITONES identified lowly detected known region-specific genes with a noisy expression pattern as spatially restricted from spatially resolved transcriptomics data. Since the spatial transcriptomics data does not have single-cell resolution, it illustrates how SEMITONES is in principle applicable to any data type where meaningful sample-to-sample similarities can be computed. Finally, we also provide functionality for the enrichment scoring of co-expression vectors, where the resulting scores may be used for the construction of co-enrichment graphs that identify regulators of cell identity (see Supplemental Materials section ‘SEMITONES for co-enrichment scoring’, Supplemental Figure S13, and Figure S14). This application will be further explored in future work. Further avenues of improvement for SEMITONES include improving its scalability in terms of memory demand for large reference cell numbers by implementing sparsity constraints on the similarity matrices, decreasing the run time for large numbers of features by improving the multiprocessing set-up, and making the significance testing procedure more efficient.

## Supplementary Material

gkac639_Supplemental_Files

## Data Availability

All datasets supporting the conclusions of this article are available from public sources. The healthy haematopoiesis scRNA-seq dataset was downloaded from the Gene Expression Omnibus (GSE139369, Accessed 28 February 2020). The healthy scATAC-seq dataset was downloaded from GitHub, accessed on 3 March 2020 (https://github.com/GreenleafLab/MPAL-Single-Cell-2019). The Mouse Brain Serial Section 1 (Sagittal-Anterior) 10x Genomics Visium spatial transcriptomics data was obtained using the SeuratData package in R (https://github.com/satijalab/seurat-data, Accessed 5 January 2021). The SEMITONES software is freely available from GitHub (https://github.com/ohlerlab/SEMITONES) under the GPL-3.0 license. The scripts and notebooks used for data processing and analyses are published on GitHub (https://github.com/ohlerlab/SEMITONES_paper).
