# Supplementary material for: Cluster-independent marker feature identification from single-cell omics data using SEMITONES
Source: Nucleic Acids Res. 2022 Jul 31;50(18):e107. doi: 10.1093/nar/gkac639 (PMC9561473; doi:10.1093/nar/gkac639)
Supplement: gkac639_Supplemental_Files [file gkac639_supplemental_files.zip › supplement.pdf]

Supplemental Results, Figures and Lists to Cluster-independent marker feature identification from single-cell omics data using SEMITONES

Anna Hendrika Cornelia Vlot[1,2], Setareh Maghsudi[3], and Uwe Ohler[1, 3, 4, *]

[1]The Berlin Institute for Medical Systems Biology, Max Delbrück Center for Molecular Medicine, Berlin, 10115, Germany
[2]Department of Computer Science, Humboldt Universität zu Berlin, Berlin, 10117, Germany
[3]Department of Computer Science, University of Tübingen, Tübingen, 72076, Germany
[4]Department of Biology, Humboldt Universität zu Berlin, Berlin, 10117, Germany
[*]Corresponding Author and Lead Contact, Uwe.Ohler@mdc-berlin.de

MATERIALS AND METHODS

SEMITONES

Reference cell selection

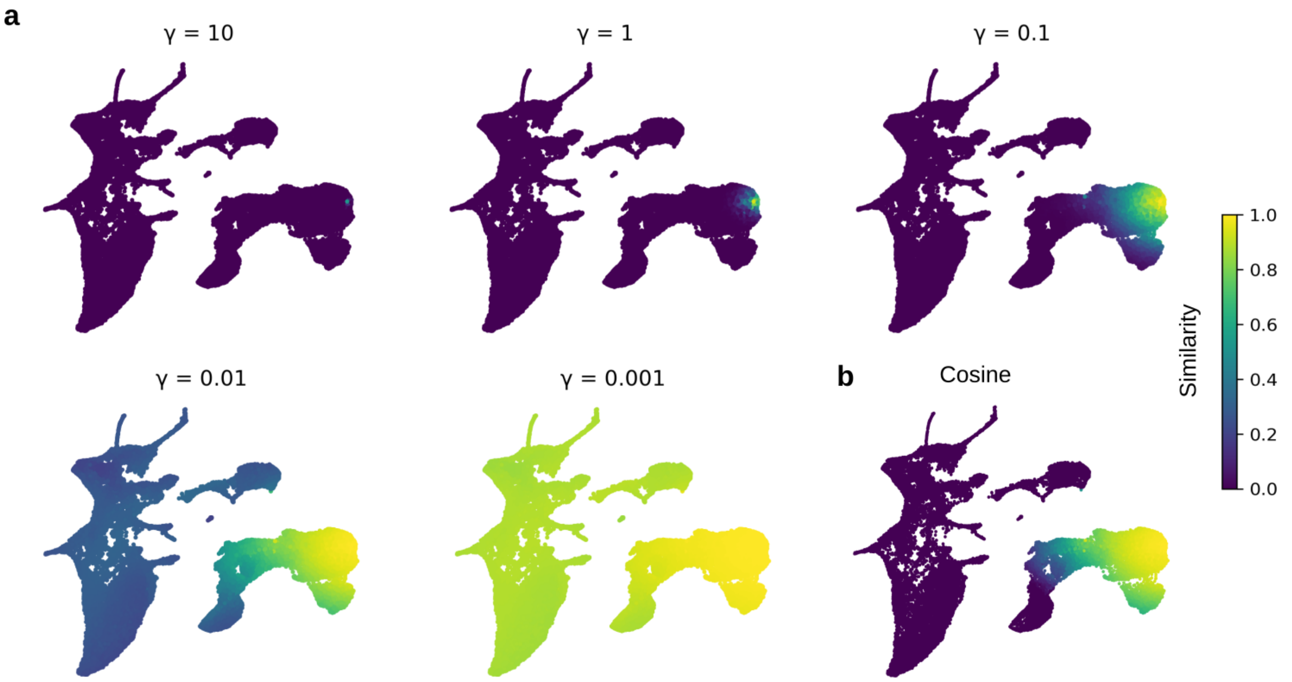

Figure S1: Illustration of similarities metrics in a 2-dimensional single-cell embedding. (a) The influence of the RBF-kernel parameter γ on the neighbourhood size considered for enrichment scoring. The plots show the similarity to a single reference cell when using an RBF-kernel to compute the similarity. **(b)** Illustration of the similarity distribution to a single cell when using the cosine kernel to compute the similarity.

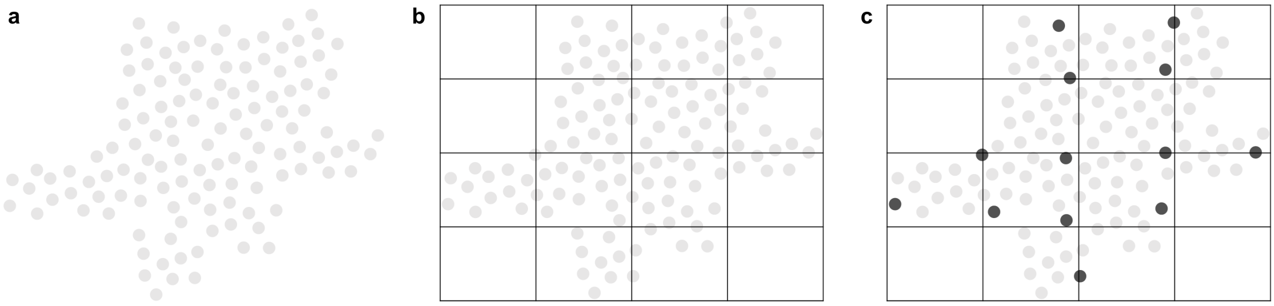

Figure S2: Illustration of the fixed-grid cell selection. Given a 2D cell embedding **(a)**, we fit a grid with a lattice of size $n \times n$ **(b)** and then select cells closest to the lattice points **(c)**.

Evaluation

Identifying marker genes with SEMITONES

List S1. Cell types selected from the CellMarker database.
AXL+SIGLEC6+ dendritic cell, Activated B cell, Activated CD4+ T cell, Activated T cell, Angiogenic T cell, Atypical memory B cell, B cell, B1 cell, Basophil, Bone marrow stem cell, CD14++CD16- monocyte, CD14+CD16+ monocyte, CD141+CLEC9A+ dendritic cell, CD16+ dendritic cell, CD1C+_A dendritic cell, CD1C+_B dendritic cell, CD1C-CD141- dendritic cell, CD4+ T cell, CD4+ T helper cell, CD4+ cytotoxic T cell, CD4+ memory T cell, CD4+ regulatory T cell, CD4+CD25+ regulatory T cell, CD4-CD28+ T cell, CD4-CD28- T cell, CD8+ T cell, CD8+ cytotoxic T cell, CD8+ regulatory T cell, Central memory T cell,

Class-switched memory B cell, Classical monocyte, Common lymphoid progenitor, Common myeloid progenitor, Conventional dendritic cell, Cytotoxic T cell, Dendritic cell, Dendritic cell progenitor, Double-negative B cell, Double-negative memory B cell, Early hematopoietic cell, Effector CD4+ memory T (Tem) cell, Effector CD8+ memory T (Tem) cell, Effector T cell, Effector memory T cell, Effector regulatory T (Treg) cell, Eosinophil, Erythroblast, Erythroid cell, Erythroid precursor, Exhausted CD4+ T cell, Exhausted CD8+ T cell, Exhausted T cell, FOXP3+ natural regulatory T (Treg) cell, Follicular B cell, Follicular T cell, Follicular dendritic cell, Follicular helper (Tfh) T cell, Foxp3+IL-17+ T cell, Germinal center B cell, Granulocyte, Granulocyte-monocyte progenitor, Hematopoietic cell, Hematopoietic precursor cell, Hematopoietic progenitor cell, Hematopoietic stem cell, IL-17Ralpha T cell, IgG memory B cell, Immature myeloid cell, Immature transitional B cell, Immune cell, Induced regulatory T (Treg) cell, Infiltrated mononuclear cell, Inflammatory cell, Intermediate monocyte, Large granular lymphocyte, Leukocyte, Lymphoblast, Lymphocyte, Lymphoid cell, Lymphoid stem cell, Lymphoid-primed multipotent progenitor, Lymphoid-primed multipotent progenitor cell, M1 macrophage, M2 macrophage, Macrophage, Marginal zone B cell, Mast cell, Mast cell progenitor, Megakaryocyte, Megakaryocyte erythroid cell, Megakaryocyte progenitor cell, Megakaryocyte-erythroid progenitor, Memory B cell, Memory T cell, Monocyte, Monocyte derived dendritic cell, Mucosal-associated invariant T cell, Multilymphoid progenitor cell, Myeloid cell, Myeloid conventional dendritic cell, Myeloid dendritic cell, Myeloid stem cell, Myeloid-derived suppressor cell, Naive B cell, Naive CD4+ T cell, Naive CD8+ T cell, Naive T cell, Naive regulatory T (Treg) cell, Natural killer T (NKT) cell, Natural killer cell, Natural memory B cell, Natural regulatory T (Treg) cell, Non-classical monocyte, Non-switched B cell, Non-switched memory B cell, Plasma cell, Plasmablast, Plasmacytoid dendritic cell, Platelet, Proerythroblast, Red blood cell (erythrocyte), Regulatory B cell, Regulatory T (Treg) cell, Responder T cell, Suppressive monocyte, Suppressor T cell, Switched memory B cell, T cell, T helper cell, T helper1 (Th1) cell, T helper17 (Th17) cell, T helper2 (Th2) cell, T helper9 (Th9) cell, T1 (Transitional) B cell, T2 (Transitional) B cell, Thymocyte, Transitional B cell, White blood cell, pro-Natural killer cell (pro-NK cell).

RESULTS

Identifying marker genes with SEMITONES

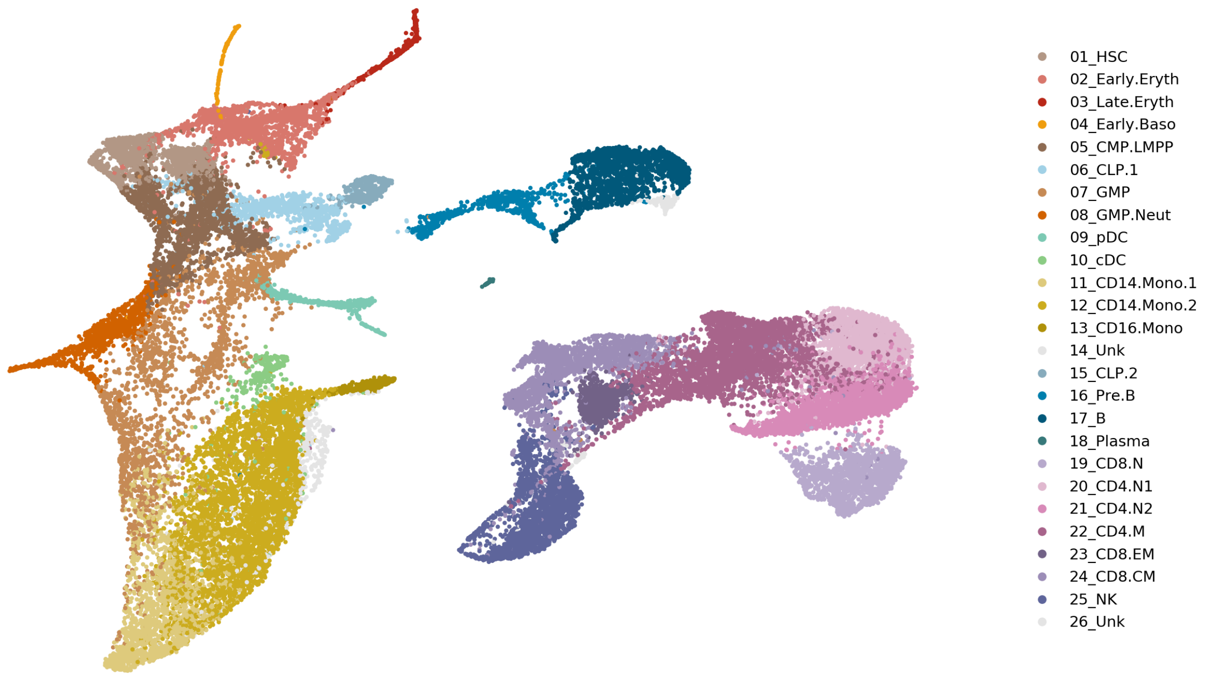

Figure S3: The cluster identities as reported in the original data publication. We visualize the original cluster identities reported in (1) on the 2-dimensional UMAP produced during processing of the scRNA-seq data for this study.

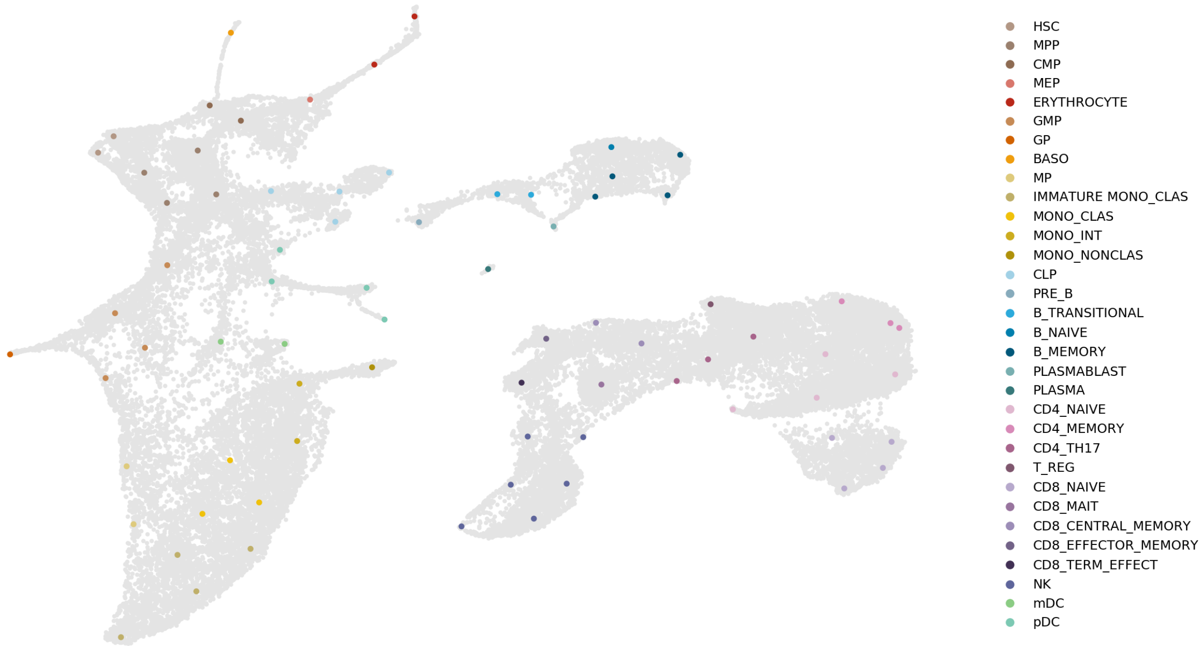

Figure S4: Manually selected reference cell annotations. Cells were selected from the two-dimensional UMAP embedding using the GUI implemented in SEMITONES.

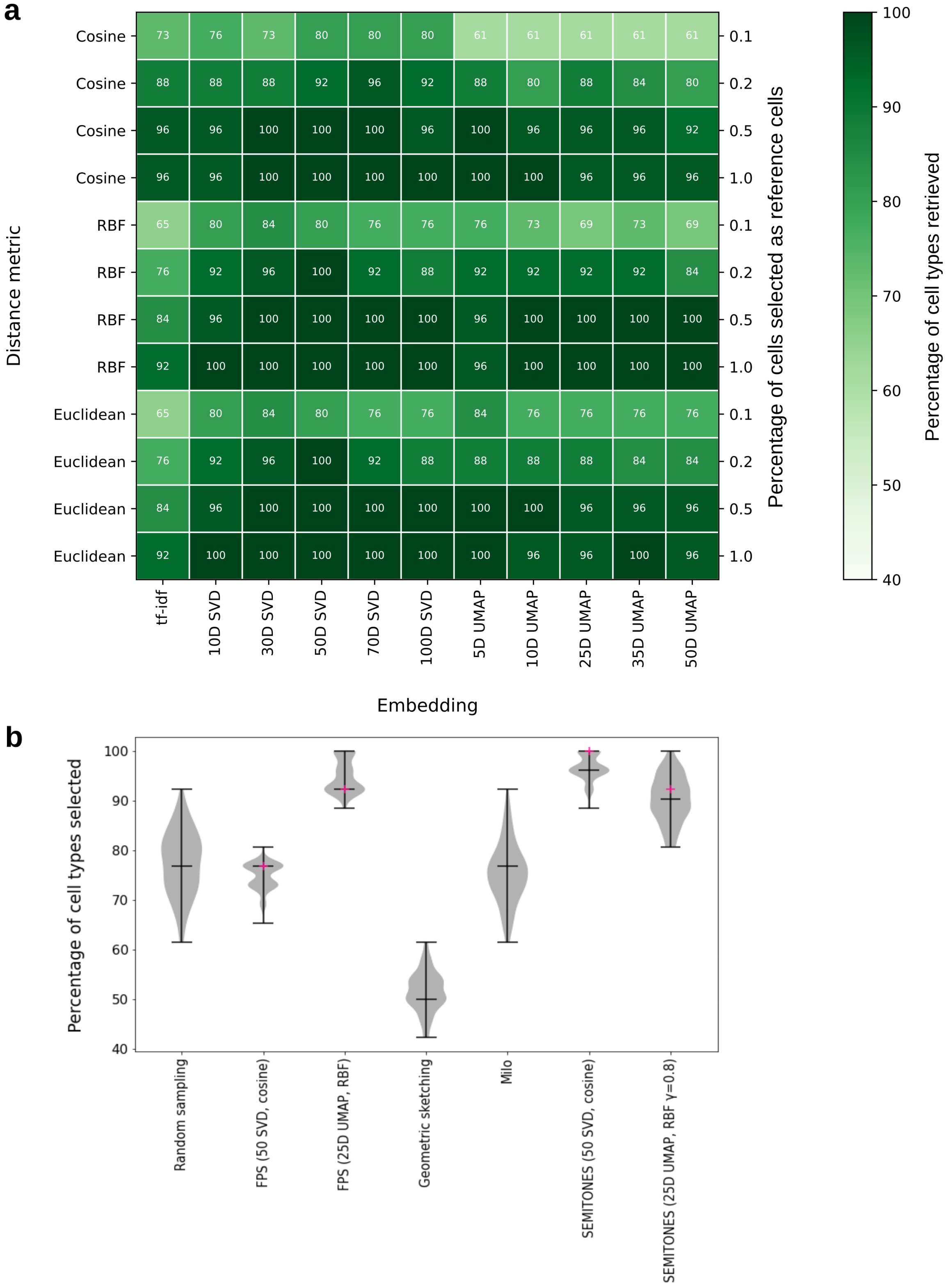

Figure S5: Performance evaluation of SEMITONES cell selection across similarity metrics, low-dimensional embeddings, and compared to alternative selection strategies. (a) The percentage of cell types, as annotated in the original data publication (1), that was retrieved using the data-driven cell selection algorithm when using specific similarity metrics over particular feature spaces. A γ-value of 0.8 was used for the RBF-kernel, as was done for all results presented in the main manuscript. In the main manuscript, we selected 0.2% of cells using a 25D UMAP. **(b)** The percentage of cell types that was retrieved using different cell selection methods. FPS stands for farthest point sampling. Geometric sketching uses the algorithm presented in (2). Milo indicates using the index cell selection method presented in the differential abundance testing package Milo (3). The pink indexers show the performance when selecting the furthest cell away from the medoid as the starting cell. In all cases, 0.2% of cells were selected as reference cells.

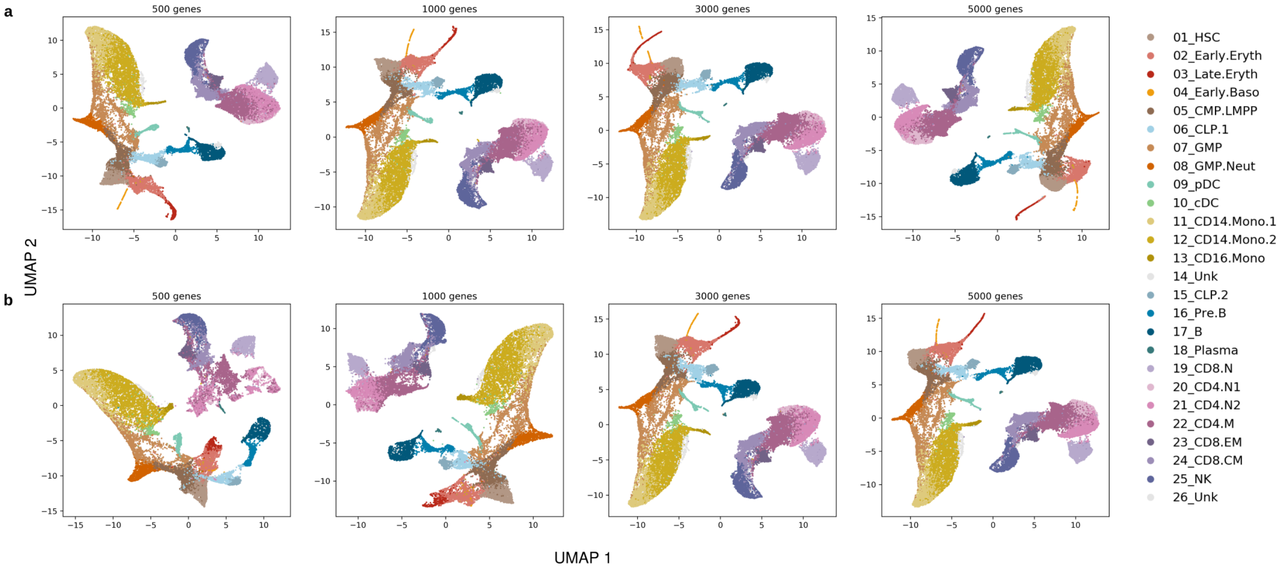

Figure S6: 2-dimensional embeddings obtained using only a few hundred or thousand top scoring SEMITONES genes. **(a)** 2-dimensional UMAPs produced with top SEMITONES genes obtained by ordering genes by the largest number of standard deviations away from the mean of the permutation null distribution. **(b)** 2-dimensional UMAPs produced with top SEMITONES genes obtained by KS-testing (see Methods).

List S2. Genes in the top 200 most significant KS-test genes but not in the top 10 most enriched genes for any reference cell.
TKTL1, RAG2, SPIB, TNFRSF13C, P4HA2, CD19, PTGDR, TBX21, MME, IRF4, CD72, PAX5, SNX22, MYL4, POU2AF1, FCRL6, XCL2, CD160, EOMES, TNFRSF13B, DNASE1L3, PRSS23, SH2D1B, S1PR5, BLNK, CRISPLD2, LAMP5, GFI1B, TTC38, BOK, CD40, COL19A1, CYB561A3, CXCL16, C5AR1, CKAP4, CD300E, CLEC4E, ANGPT1, CLIC3, B3GAT1, CD163, KLHL14, PILRA, LILRA5, STAG3, APMAP, QPCT, ABCB4, ASGR2, LRP1, FCRL5, SPTA1, RAG1, SLC7A7, RTN1, APOBEC3A, ZNF385D, BEX1, PPP1R14A, STAB1, HK3, FAM198B, C19orf38, DYSF, CD93, MAFB, HPGDS, BPI, HLA-DOB, SLC46A2, ASPM, MEGF9, RXRA, NCF2, TNFAIP2, ZWINT, WLS, CSF1, LINC00937, LRRK2, GNS, KCTD12, G0S2, CRYM, PNOC, LDLRAD4, CREB5, BRI3, RASD1, ASGR1, CFP, CDH1.

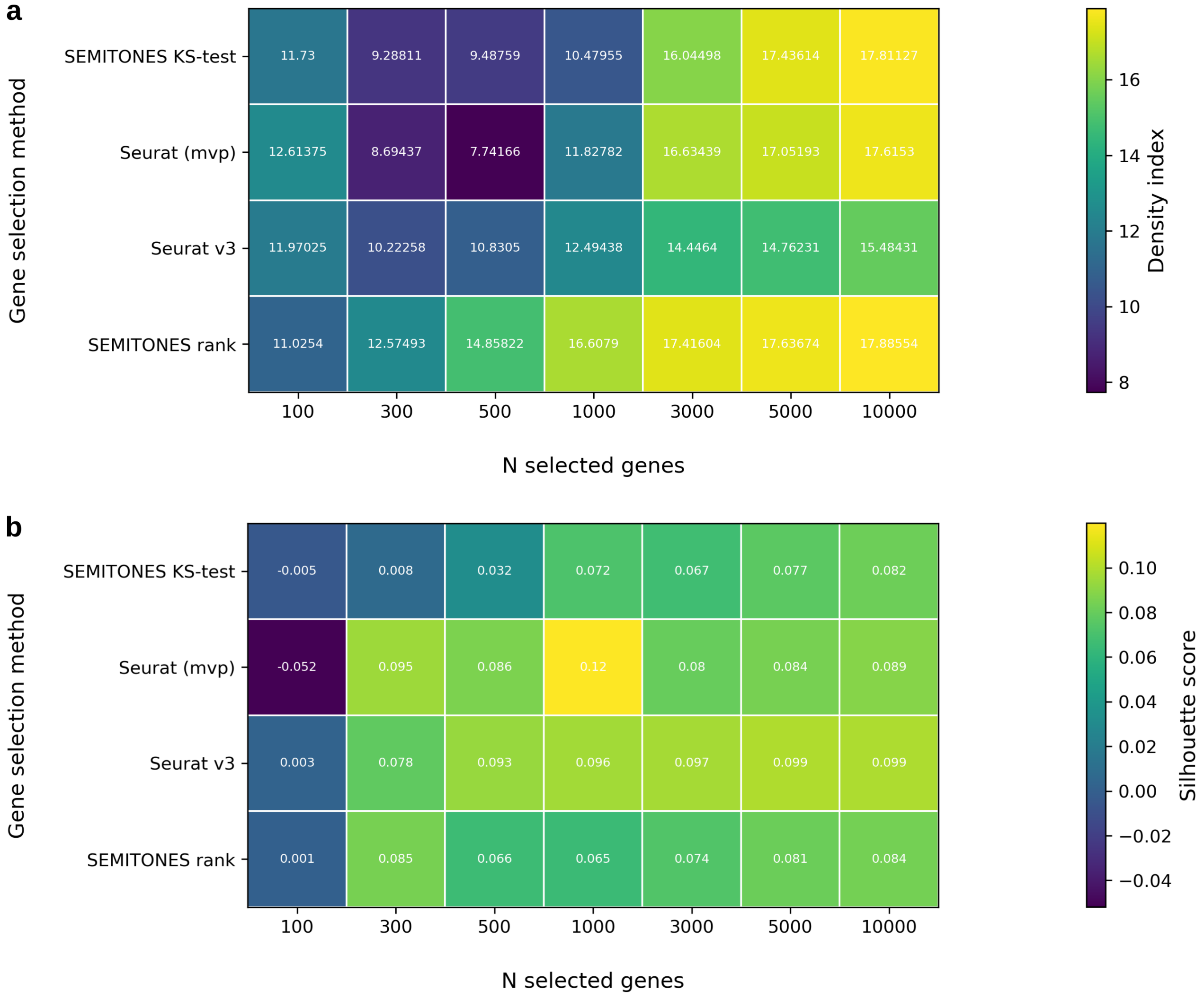

Figure S7: Density index and silhouette scores of over 50-dimensional reduced embedding of scRNA-seq healthy heamatopoiesis data. **(a)** The density index as taken from (4) computed over the top 50 SVD components when selecting a certain number of top scoring genes using either SEMITONES KS-testing, Seurat v2's mvp, Seurat v3's variable gene selection, or SEMITONES rank-based gene selection. **(b)** The silhouette scores computed over the top 50 SVD components using the Euclidean distance as a distance metric (as is the default used in sklearn.metrics.silhouette_score), obtained using the top "N selected genes", using the cluster annotations from the original data publication (1).

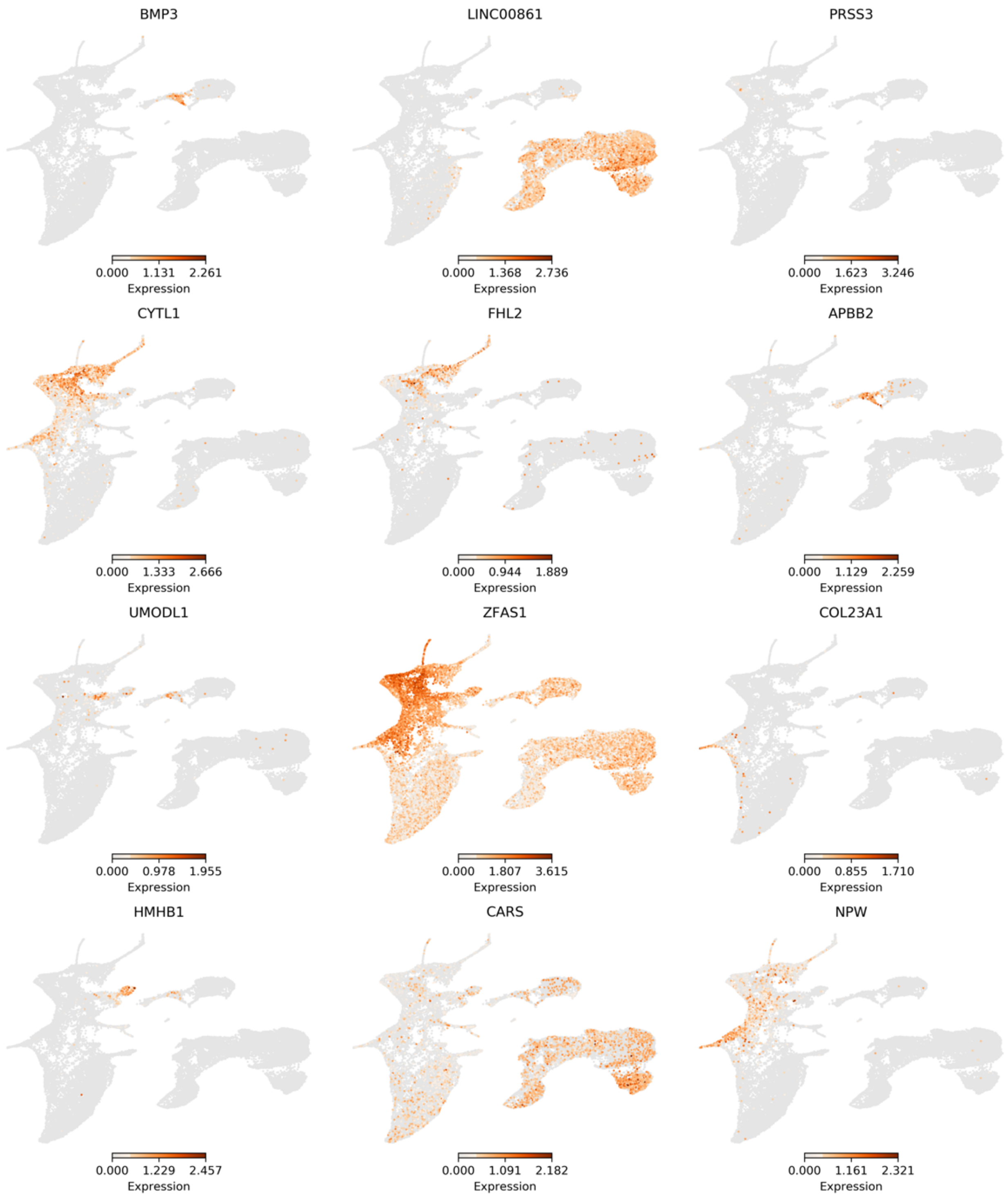

Figure S8: High scoring SEMITONES genes without corresponding literature evidence show marker like expression profiles. The expression profile of genes that are in the top 10 genes for any reference for which no literature or database evidence was found.

Neighbourhood-specific *cis*-regulatory element identification

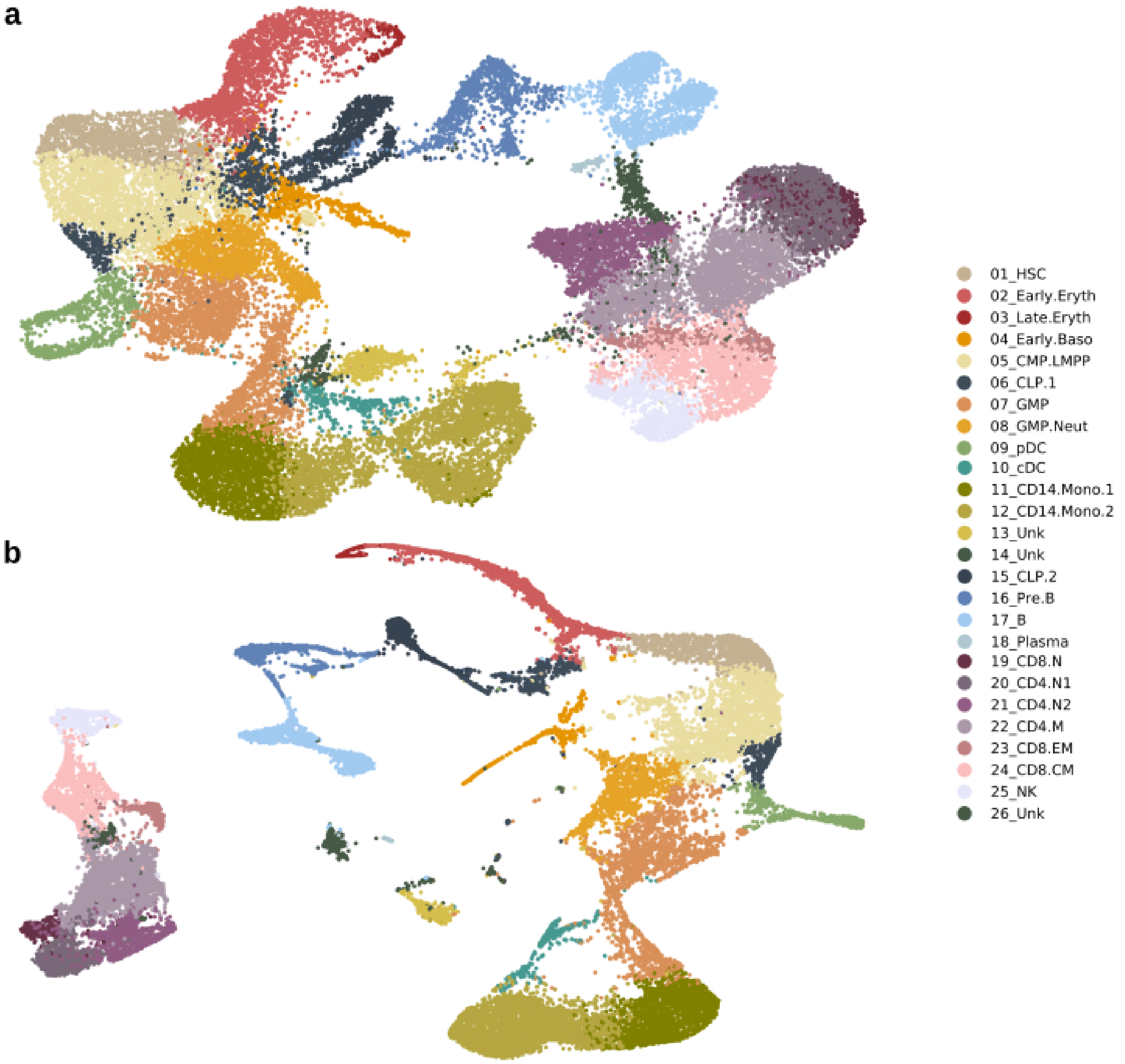

Figure S9: Two-dimensional embeddings improve when performing dimensionality reduction using only SEMITONES selected informative peaks. UMAP representations of scATAC-seq data used in this study using all peaks **(a)** versus only the top scoring peaks **(b)**.

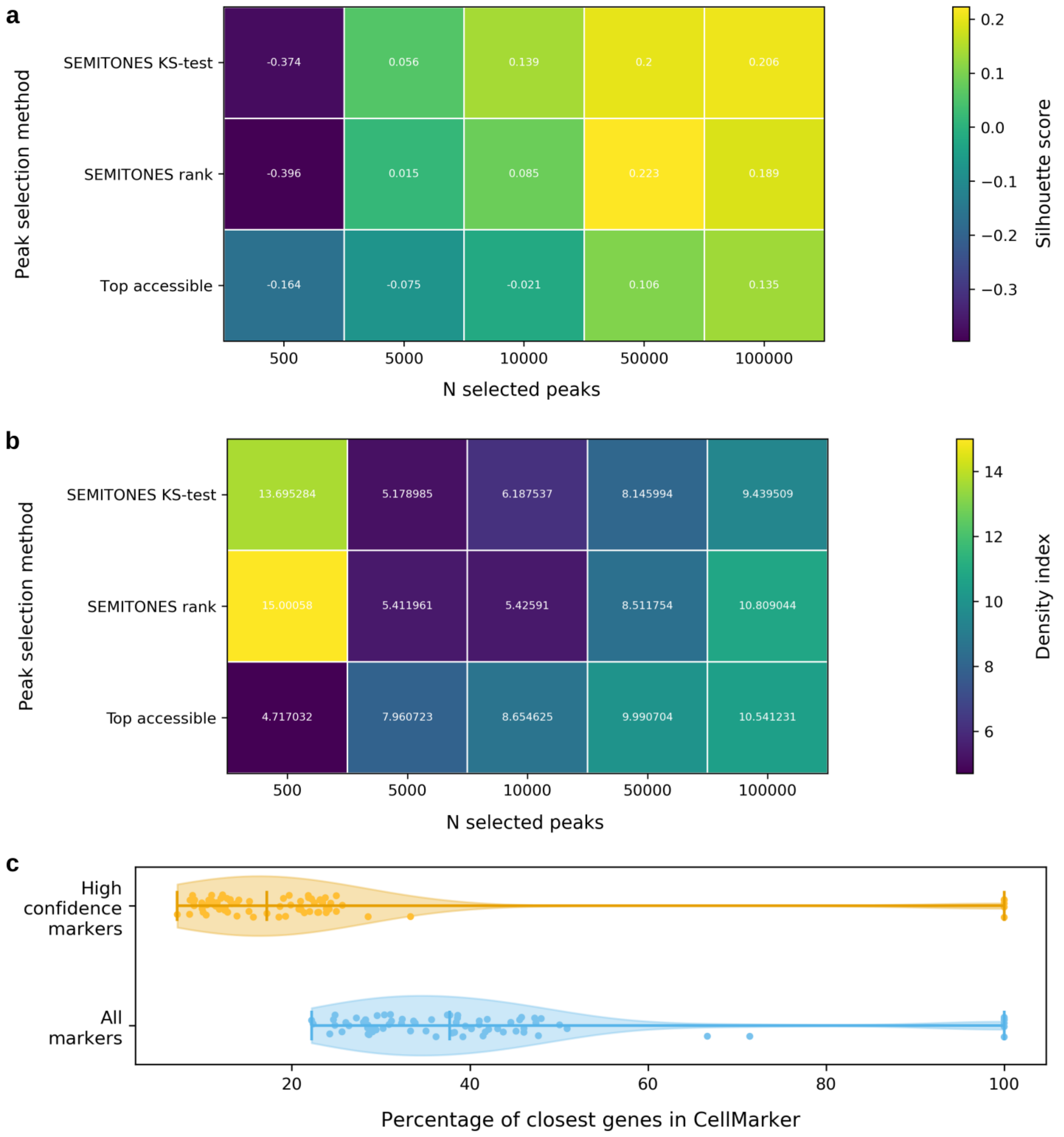

Figure S10: Density index and silhouette scores of over 50-dimensional reduced embedding of scRNA-seq healthy heamatopoiesis data. **(a)** The density index as taken from (4) computed over the top 50 SVD components when selecting a certain number of top scoring peaks using SEMITONES KS-testing, SEMITONES rank-based gene, or simply taking the top accessible peaks. **(b)** The silhouette scores computed over the top 50 SVD components, obtained using the top "N selected genes", using the cluster annotations from the original data publication (1). **(c)** The distribution of the percentage of nearest genes to significantly enriched regions obtained by SEMITONES that are found as a marker in the CellMarker database that are supported by 3 or more sources (high confidence, orange) or 1 or more sources (all markers, blue).

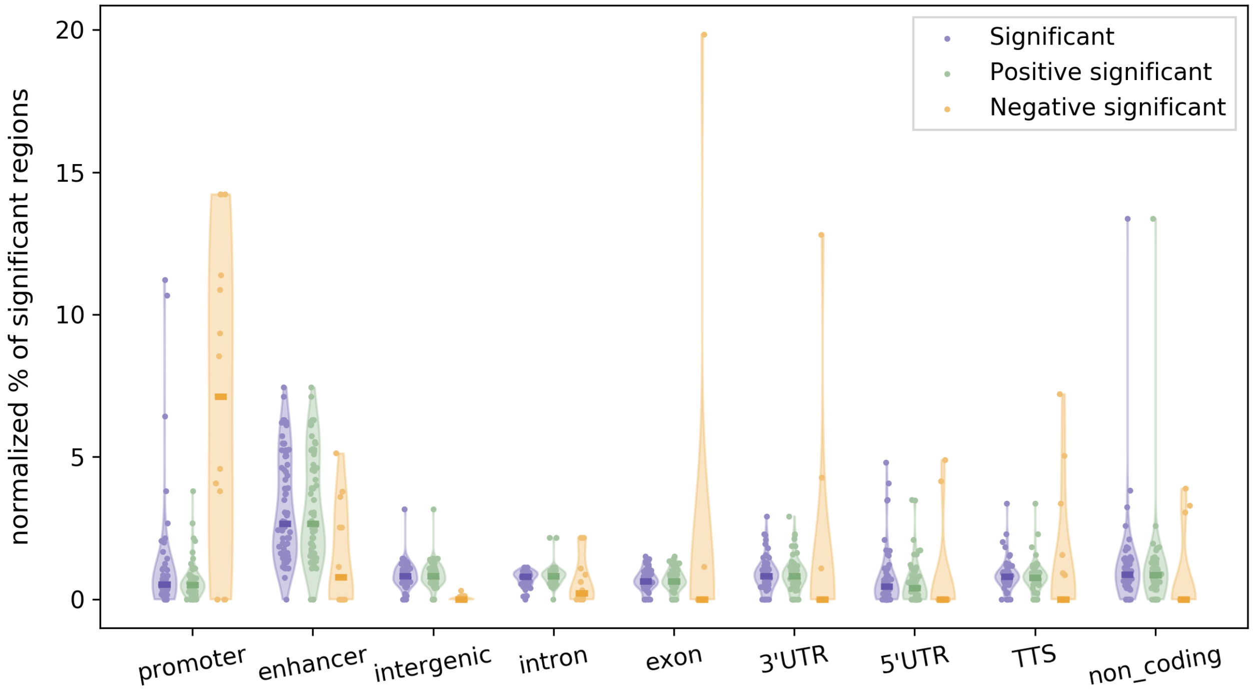

Figure S11: Normalized percentage of peaks with a certain annotation. This figure illustrates the distribution of the percentages of all, significantly enriched and selectively accessible, and significantly enriched and selectively inaccessible regions with particular HOMER annotations, or an enhancer annotation in FANTOM5. Values are normalized for the total number of regions that have a certain annotation.

SEMITONES identifies spatially restricted genes

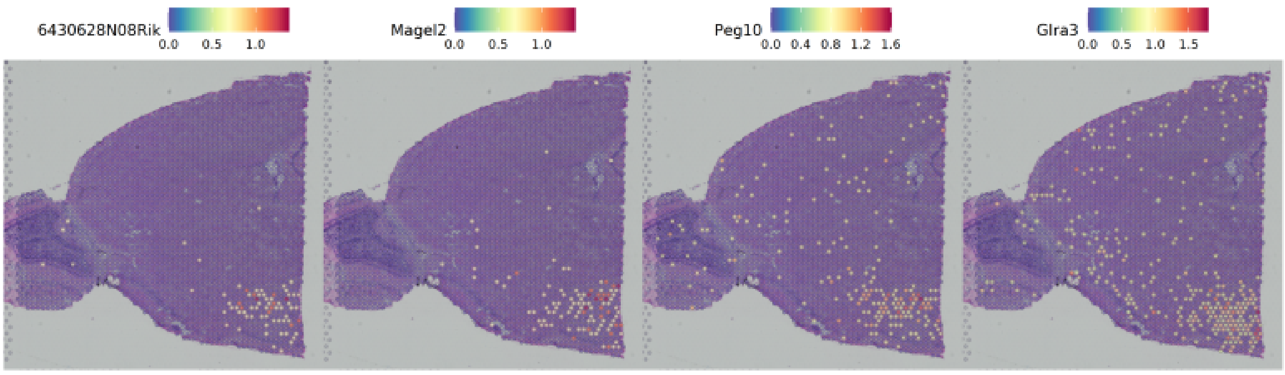

Figure S12: Spatially restricted genes only identified by SEMITONES. The spatially resolved expression profile of 4 genes that were identified as being in the top 100 enriched genes by SEMITONES, but were not identified as significantly spatially variable by the variogram method as implemented in Seurat v3.

SEMITONES for co-enrichment scoring

a

	g1	g2	g3	g4	g5	g6	g7	g8	g9	g10
**c1**	0	3.26	2.89	1.61	2.48	2.08	1.39	3.22	2.4	2.4
**c2**	2.48	0	0	0	0	0	0	0	3.09	2.08
**c3**	0	2.4	3.3	1.61	0	1.79	0	0	0	0
**c4**	3.22	0	0	0	0	3.04	0	2.94	0	2.83
**c5**	0	3.09	1.95	2.89	0.69	0	1.1	0	0	0
**c6**	2.94	2.77	2.71	2.64	2.08	3.3	0	3	3.26	2.48
**c7**	0	0	0	0	3.4	0	1.61	0	2.3	0
**c8**	1.61	0	0	3.09	0	3.3	0	3.22	0	3.4
**c9**	0	0.69	2.89	1.1	2.89	1.95	0	0	2.77	2.56
**c10**	1.39	0	2.48	0	0	0	0	3	0	1.1
**c11**	3	0	1.79	3	0	0	0	2.08	1.39	0
**c12**	0.69	2.4	0	2.94	3.26	3.14	0.69	1.1	3.4	3.22
**c13**	0	3.33	0	0	0	3.4	2.94	3.18	0	3.4
**c14**	3.37	0	2.77	0	2.94	0	0	0	2.48	0
**c15**	0	0	3.04	3.26	0	3.33	2.89	0	0	0

×

b

	g1-g2
**c1**	0
**c2**	0
**c3**	0
**c4**	0
**c5**	0
**c6**	8.14
**c7**	0
**c8**	0
**c9**	0
**c10**	0
**c11**	0
**c12**	1.66
**c13**	0
**c14**	0
**c15**	0

SEMITONES

c

	g1	g2	g3	g4	g5	g6	g7	g8	g9	g10
**g1**										
**g2**	0.01									
**g3**	5e-4	0.26								
**g4**	0.22	0.42	1e-3							
**g5**	0.56	0.09	.95	0.29						
**g6**	0.02	2e-2	.01	0.46	1e-5					
**g7**	8e-6	23e-6	1e-6	0.01	0.43	0.01				
**g8**	0.20	1e-3	0.42	2e-4	1-e3	.43	0.36			
**g9**	0.72	0.69	21e-5	0.53	0.92	5e-7	0.15	2e-5		
**g10**	0.48	15e-6	0.39	0.75	0.07	7e-4	1e-3	0.61	0.72	

Figure S13: SEMITONES for computation of co-enrichment scores to construct co-enrichment graphs. (a) An example log-transformed gene expression matrix of cells (c1, ..., c15) by genes (g1, ..., g10). **(b)** The co-expression vector of gene g1 and gene g2 when using the multiplication strategy to construct co-expression vectors, comparable to defining an interaction term in a multiple regression model. **(c)** The resulting co-enrichment score matrix for all genes (g1, ..., g2) that serves as a an adjacency matrix which represents the co-enrichment graph.

Given vectors that represent some form of co-expression or co-occurrence of features, SEMITONES can also compute co-enrichment scores (see Supplemental Figure 13). For this, one first needs to construct these co-expression or co-occurrence vectors from the individual feature vectors in the cell by feature matrix. In Supplemental Figure 13ab, we illustrate this for two genes (g1 and g2), we simply multiple the individual feature vectors equivalent to using an interaction term in multiple regression. In SEMITONES, we provide 3 more ways to represent a set of two or more continuous features (i.e. gene expression vectors) in a single co-expression vector, namely 1) selecting the highest expression value in the feature set as a representative value per cell, 2) selecting the lowest expression value in the feature set as a representative value per cell, and 3) using the median expression value of the feature set as a representative value per cell. For binary feature vectors, like for accessibility profiles in scATAC-seq, SEMITONES implements a strategy to either take the median value (4), or setting the entry per cell to 0 (i.e. absent) if neither of the features is detected and to 1 (i.e. present) if either or both of the features is detected in that cell. Applying SEMITONES to these co-expression or co-occurrence vectors provides co-enrichment scores for each feature pair in each reference cell. For each reference cell, these scores can be organized in a gene by gene matrix that serves as an adjacency matrix that represents a co-enrichment graph (Figure 13c). In the resulting co-enrichment graphs, features (i.e. vertices) are connected by edges weighted by the co-enrichment scores. Thus, the weight of the edges indicates how strongly the combination of two features is enriched in a particular neighbourhood, i.e. how selective the combined expression of two genes is for the reference cell neighbourhood.

To illustrate the application of SEMITONES for co-enrichment scoring and co-enrichment graph construction, we apply SEMITONES to the healthy
haematopoiesis scRNA-seq data also used in the main body of the SEMITONES manuscript. In the examples outlined below, we chose to construct co-expression vectors by taking the maximum value of each gene in each gene set. For ease of interpretation of the results, we only perform enrichment scoring in a single reference cell per *de novo* annotated cell state. If there are several reference cells of the same annotation, the cell in which the primary cell state marker is most highly expressed was selected. Besides, for computational efficiency, we compute co-enrichment scores for gene sets of genes that are significantly enriched (at 25 standard deviations away from the mean of the permutation null distribution) in at least one reference cell in the subset, resulting in 333974 gene pairs. We compute co-enrichment scores for these gene sets in each reference cells, and construct co-enrichment graphs in which we eliminate any edges with corresponding co-enrichment scores 30 or less standard deviations away from the mean of the permutation null. To highlight the most important interactions and to enable comprehensive visualization, we then compute the maximum spanning tree (MST) using networkx v2.4 (5). The regulatory potential of each gene is characterized by its current flow betweenness centrality which quantifies how essential a specific gene is for the flow of information in the network. The visualizations are produced using the Netwulf package in Python (6).

Inspection of some co-enrichment graphs reveals many known gene interactions from haematopoietic development. For example, the co-enrichment graph of naive CD8+ T cells reveals interactions but *CD3E*, *CD3D*, and *CD8A* genes (see Figure 14a), which code for constituents of the CD3-complex and the cytotoxic T cell surface marker (CD8). It is suggested that the function of CD8 is to transport the Lck protein to the CD3-complex during T cell development (7), making it likely that these genes would be co-enriched in naive CD8+ cells. Additionally, the STRING v11 database contains predicted interactions between the protein products of these genes (8). Likewise, in transitional B cells, we observe strong co-enrichment of *IGLL5* and *CD79B* (see Figure 14b), which respectively encode the Igβ and Igλ proteins that are involved in pre-B cell receptor signalling, a central process in the development of immature B cells (9). Besides, many genes with high current flow betweenness centrality values in the co-enrichment graph MSTs include known regulators of cell identity. To illustrate, in erythrocyte co-enrichment graphs, we identify the well known regulators *GFI1B* and *HES6* (10; 11) among the top 10 most central vertices. Individually, these genes rank 31st and 46th in the erythrocyte neighbourhood, illustrating how co-enrichment scores help to identify important regulators of cell identity even when these are not the most selectively expressed genes in a particular neighbourhood. Similarly, the highly central *S100A4* gene in the Th17 co-enrichment graph (see Figure S13c) is only the 52nd most enriched gene in the Th17 neighbourhood. This rank is intuitively coherent with the high expression in cells outside of the Th17 population, in particular in monocytes, myeloid/monocytic dendritic cells (mDC), natural killer (NK) cells, and all other mature T cell populations. Interestingly, a potential role of *S100A4* in Th17 cell differentiation has been suggested before, albeit in Rheumatoid Arhritis mouse models (12). Taken together, these results exemplify the potential of SEMITONES for the identification biologically meaningful regulatory interactions from scRNA-seq data.

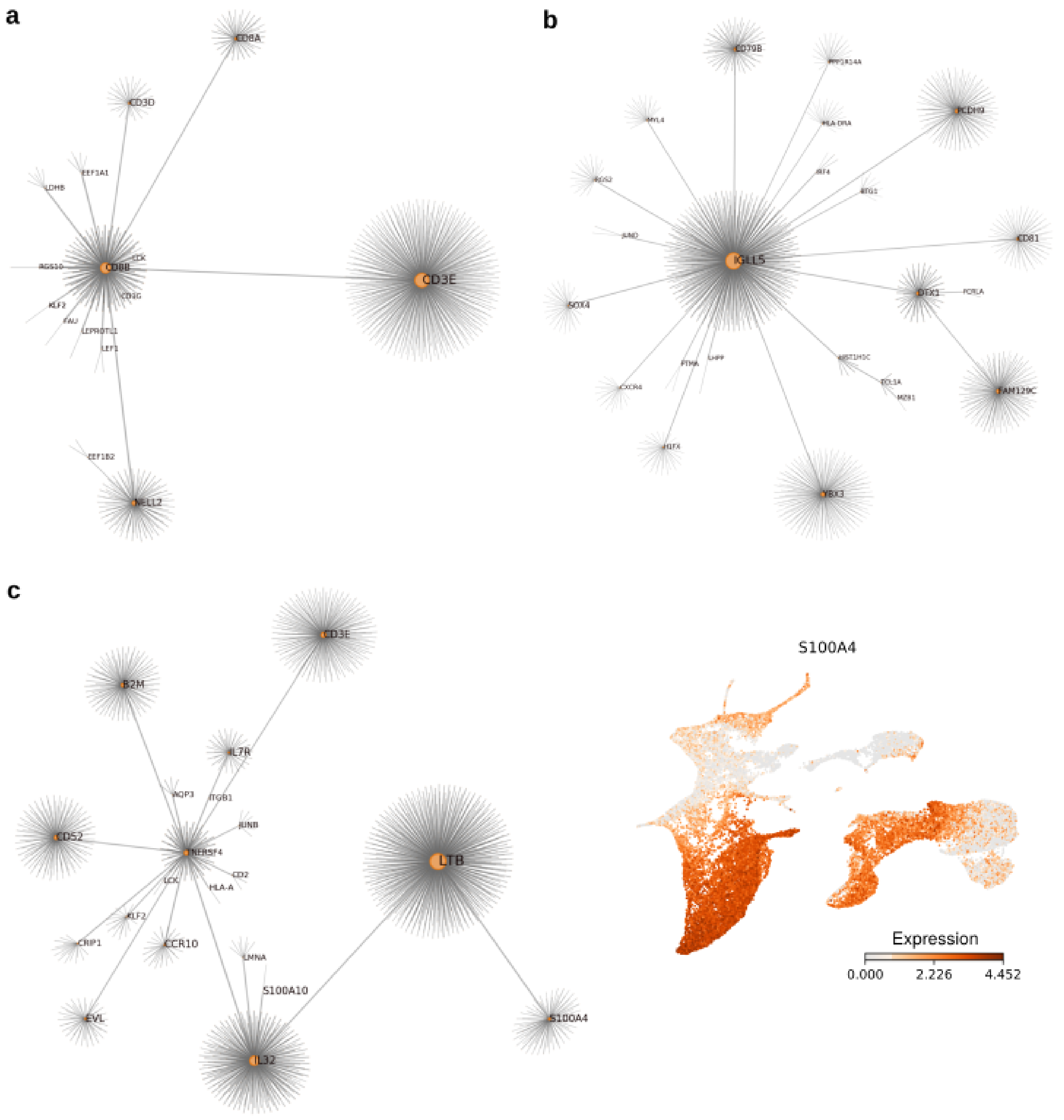

Figure S14: Maximum spanning tree representations of co-enrichment graphs constructed by SEMITONES. (a) The maximum spanning tree (MST) of the naive CD8+ T cell co-enrichment graph. **(b)** The MST of the transitional B cell co-enrichment graph. **(c)** The MST of the Th17 cell co-enrichment graph shows a central role the the *S100A4* gene which has a non-Th17-specific expression pattern. In all graphs, the vertex size is proportional to their weighted degree.

References

[1] Granja JM, Klemm S, McGinnes LM, Kathiria AS, Mezger A, Corces MR, et al. Single-cell multiomic analysis identifies regulatory programs in mixed-phenotype acute leukemia. Nature Biotechnology. 2019;37:1458-65.

[2] Hie B, Cho H, DeMeo B, Bryson B, Berger B. Geometric Sketching Compactly Summarizes the Single-Cell Transcriptomic Landscape. Cell Systems. 2019;8(6):483-93.e7. Available from: https://www.sciencedirect.com/science/article/pii/S2405471219301528.

[3] Dann E, Henderson NC, Teichmann SA, Morgan MD, Marioni JC. Differential abundance testing on single-cell data using k-nearest neighbor graphs. Nature Biotechnology. 2021.

[4] Ranjan B, Sun W, Park J, Mishra K, Schmidt F, Xie R, et al. DUBStepR is a scalable correlation-based feature selection method for accurately clustering single-cell data. Nature Communications. 2021;12:5849.

[5] Hagberg SDA A A, Swart PJ. Exploring network structure, dynamics, and function using NetworkX. In: Proceedings of the 7th Python in Science Conference (SciPy2008); 2008. p. 11–15.

[6] Aslak U, Maier BF. Netwulf: Interactive visualization of networks in Python. Journal of Open Source Software. 2019;4(42):1425.

[7] Artyomov MN, Lis M, Devadas S, Davis MM, Chakraborty AK. CD4 and CD8 binding to MHC molecules primarily acts to enhance Lck delivery. Proceedings of the National Academy of Sciences. 2010;107:16916 16921.

[8] Szklarczyk D, Gable AL, Lyon D, Junge A, Wyder S, Huerta-Cepas J, et al. STRING v11: protein–protein association networks with increased coverage, supporting functional discovery in genome-wide experimental datasets. Nucleic Acids Research. 2019;47:D607 D613.

[9] Wang LD, Clark MR. B-cell antigen-receptor signalling in lymphocyte development. Immunology. 2003;110:411-20.

[10] Vassel L, Beauchemin H, Lemsaddek W, Krongold J, Trudel M, Möröy T. Growth factor independence 1b (gfi1b) is important for the maturation of erythroid cells and the regulation of embryonic globin expression. PLoS One. 2014;9(5):e96636.

[11] da Cunha AF, Brugnerotto AF, Duarte ASS, Lanaro C, Costa GGL, Saad STO, et al. Global gene expression reveals a set of new genes involved in the modification of cells during erythroid differentiation. Cell proliferation. 2010;43:297-309.

[12] Brisslert M, Bian L, Svensson MND, Santos RMF, Jonsson IM, Barsukov IL, et al. S100A4 regulates the Src-tyrosine kinase dependent differentiation of Th17 cells in rheumatoid arthritis. Biochimica et biophysica acta. 2014;1842:2049-59.

Supplemental Table 1. Top 10 most highly enriched genes.

ID	Annotation	Top 10 most highly enriched genes
282	B_MEMORY	MS4A1, LINC00926, BANK1, CD79A, HLA-DQB1,FCER2, HLA-DQA1, CD22, FCRL2, ADAM28
855	MONO_CLAS	VCAN, LYZ, FCN1, S100A9, S100A8, CD36, GRN, FOSL2, CST3, MNDA
908	GMP	MPO, ELANE, PRTN3, PRSS57, AZU1, MGST1, C1QTNF4, MS4A3, CTSG, NUCB2
1223	CD4_MEMORY	ADTRP, KLF2, JUNB, LTB, AP3M2, IL7R, TPT1, LEPROTL1, TRABD2A, EEF1A1
1772	CD4_MEMORY	LTB, JUNB, KLF2, AP3M2, TNFAIP3, LEPROTL1, TPT1, ADTRP, EEF1A1, CD3E
2628	MPP	SPINK2, EGFL7, CD34, CDK6, PRSS57, MDK, LAPTM4B, BAALC, C1QTNF4, AC002454.1
2680	pDC	LILRA4, CLEC4C, IL3RA, SMPD3, SERPINF1, PLD4, PTPRS, SCT, PTCRA, IRF7
2940	MP	RETN, RNASE2, LYZ, GRN, LGALS1, S100A8, SRGN, CAPG, AZU1, MS4A6A
3447	IMMATURE MONO_CLAS	S100A12, PLBD1, S100A8, S100A9, PADI4, RBP7, FCN1, FOLR3, CDA, HP
4672	GP	CTSG, PRTN3, ELANE, AZU1, MPO, RNASE3, COL23A1, PRSS57, CLEC11A, MS4A3
4927	IMMATURE MONO_CLAS	S100A12, PLBD1, S100A8, S100A9, FCN1, PADI4, FOLR3, VCAN, RBP7, CDA
5577	ERYTHROCYTE	AHSP, HBB, CA1, HBQ1, RHAG, GYPA, NMU, HBA1, EPCAM, KCNH2
5631	CD4_TH17	LTB, TNFRSF4, TNFAIP3, KLF2, JUNB, AQP3, CD3D, GPR183, IL32, ICOS
5811	IMMATURE MONO_CLAS	S100A8, S100A9, S100A12, FCN1, VCAN, FOLR3, PLAUR, LYZ, PLBD1, CTSD
6116	PRE_B	KIFC1, TOP2A, NUSAP1, BIRC5, RRM2, MYBL2, AURKB, UBE2C, MKI67, CDC20
6126	CD8_EFFECTOR_MEMORY	GZMK, DUSP2, CCL5, CD8A, CST7, GZMA, CMC1, CCL4, CD8B, GZMM
6903	CD8_NAIVE	CD8B, CD8A, NELL2, CD3D, LEPROTL1, LEF1, MGAT4A, CD3E, EEF1A1, CCR7
6981	CD4_MEMORY	KLF2, LTB, TPT1, CD3E, LEPROTL1, EEF1A1, CD3D, CD7, JUNB, CD3G
7059	CD4_MEMORY	KLF2, LTB, LEPROTL1, TPT1, CD3E, EEF1A1, JUNB, CD7, ADTRP, CD3D
7284	B_TRANSITIONAL	DTX1, IGLL5, BMP3, TCL1A, CD79B, APBB2, BCL7A, NEIL1, TCL1B, HRK
7900	CD4_TH17	TNFRSF4, IL7R, LTB, IL32, ITGB1, TRADD, AQP3, TNFAIP3, JUNB, KLF2
8089	CD8_TERM_EFFECT	NKG7, GZMH, CCL5, GZMA, CD8A, CST7, CMC1, CCL4, KLRG1, CTSW
8560	CD8_CENTRAL_MEMORY	GZMK, CCL5, DUSP2, LYAR, ZNF683, IL32, CD8A, CD8B, CXCR3, IL7R
9687	CD4_TH17	TNFRSF4, IL32, ITGB1, LTB, AQP3, IL7R, CRIP2, TNFRSF18, RORA, CD3D
11487	B_NAIVE	LINC00926, FCER2, MS4A1, CD79A, TCL1A, BANK1, FCRL1, HLA-DQB1, HVCN1, HLA-DQA1
11793	CD4_MEMORY	LTB, KLF2, JUNB, CD3E, TPT1, LEPROTL1, CD7, CD3D, EEF1A1, FHIT
11816	MONO_CLAS	VCAN, S100A9, S100A8, FCN1, LYZ, S100A12, CD36, CD14, PLAUR, MNDA
12044	CD8_NAIVE	CD8B, CD8A, NELL2, REG4, CD248, CD3D, CD7, CD3E, MGAT4A, LEPROTL1
12527	CLP	DNTT, CYGB, IGLL1, VPREB1, ADA, UMODL1, EBF1, ZCCHC7, LAT2, HMGB1
13295	GMP	ELANE, PRTN3, MPO, AZU1, CTSG, MS4A3, PRSS57, C1QTNF4, MGST1, NUCB2
13665	pDC	SCT, IRF8, CCDC50, TCF4, UGCG, IL3RA, ITM2C, LGMN, LILRA4, APP
13820	HSC	AVP, CRHBP, EGFL7, MDK, DDX3Y, MEG3, CD34, SPINK2, RBPMS, NPR3
14062	HSC	PRSS1, SPINK2, PRSS3, EGFL7, AVP, CD34, BAALC, CRHBP, TSC22D1, LAPTM4B

Supplemental Table 1. Top 10 most highly enriched genes.

14595	HSC	EGFL7, SPINK2, AVP, CD34, MDK, CRHBP, ZFAS1, NPR3, LAPTM4B, CDK6
15070	BASO	CLC, HDC, PRG2, MS4A2, MS4A3, LMO4, TPSAB1, EPX, GATA2, SLC45A3
15131	HSC	AVP, CRHBP, MEG3, SPINK2, ANKRD28, C6orf48, NRIP1, MYCT1, CYTL1, HTR1F
15806	CMP	CNRIP1, GATA2, ITGA2B, CYTL1, GATA1, PRKAR2B, FCER1A, KLF1, PBX1, C2orf88
15914	GMP	MPO, C1QTNF4, PRSS57, NUCB2, IGFBP7, FABP5, MGST1, NAP1L1, NPW, IGLL1
15952	MPP	SPINK2, EBPL, C6orf48, CDK6, NAP1L1, ZFAS1, NACA2, CYTL1, BTF3, ANKRD28
16578	MPP	SPINK2, C6orf48, CRHBP, AVP, NACA2, EBPL, HOPX, ANKRD28, ZFAS1, CYTL1
17140	MPP	SPINK2, AC002454.1, C1QTNF4, IGFBP7, NAP1L1, NUCB2, IGLL1, CDK6, FABP5, EBPL
17720	MEP	UBE2C, CENPF, CKS1B, TOP2A, HMMR, NUSAP1, CKS2, CDKN3, TYMS, CKAP2L
18416	MEP	HBD, APOC1, CNRIP1, KLF1, FHL2, NECAB1, PRKAR2B, ITGA2B, GATA1, APOE
19106	CLP	AKAP12, ARPP21, VPREB1, DNTT, CD9, VPREB3, HMHB1, LINC01013, LINC00114, CD79B
19931	mDC	FCER1A, IGSF6, CTSV, ENHO, ANXA2, H2AFZ, LGALS1, HLA-DPA1, CLSPN, TUBA1B
21039	CD4_TH17	IL7R, TNFRSF4, IL32, AQP3, LTB, ITGB1, RORA, TRADD, TNFRSF18, TNFRSF25
21476	MONO_NONCLAS	CDKN1C, HES4, C1QA, MS4A7, FCGR3A, TCF7L2, HMOX1, LYPD2, IFITM3, CSF1R
23908	MONO_CLAS	CD14, CTSS, SERPINA1, LGALS2, TYMP, MNDA, S100A9, VCAN, SAT1, NEAT1
24119	NK	FGFBP2, GNLY, GZMH, KLRF1, PRF1, GZMB, KLRD1, NKG7, SPON2, FCGR3A
24344	IMMATURE MONO_CLAS	S100A12, S100A9, S100A8, CD14, VCAN, SLC11A1, MNDA, RGS2, NAMPT, CTSS
24711	MONO_INT	LGALS2, CTSS, TYMP, NEAT1, CD14, SERPINA1, SAT1, S100A9, FGL2, TYROBP
25197	mDC	CLEC10A, ENHO, CD1C, PKIB, FCER1A, CD1E, HLA-DQA1, HLA-DQB1, HLA-DRB5, CPVL
25398	CD4_NAIVE	LEF1, TCF7, IL7R, CD3G, NOSIP, CD3E, PIK3IP1, CD3D, AES, TPT1
26206	B_MEMORY	MS4A1, IGLL5, CD79A, BANK1, LINC00926, HLA-DQB1, HLA-DQA1, FCER2, P2RX5, BLK
26333	MONO_CLAS	CD14, VCAN, S100A9, S100A8, S100A12, MNDA, CTSS, SLC11A1, TYMP, FCN1
26382	CD8_MAIT	KLRB1, SLC4A10, GZMK, LTK, NCR3, KLRG1, DUSP2, LYAR, PRR5, RORC
26667	CD8_MAIT	KLRB1, GZMK, SLC4A10, KLRG1, LTK, DUSP2, NCR3, LYAR, GZMA, CCL5
26730	MONO_CLAS	CD14, CTSS, S100A9, MNDA, VCAN, SLC11A1, S100A8, TYMP, SERPINA1, S100A12
27046	NK	GNLY, KLRF1, PRF1, KLRD1, NKG7, GZMH, CTSW, GZMB, FGFBP2, GZMA
27075	NK	GNLY, FGFBP2, GZMH, KLRF1, PRF1, KLRD1, NKG7, GZMB, FCGR3A, SPON2
27644	CD4_NAIVE	LEF1, TCF7, CCR7, NOSIP, PIK3IP1, CD3E, TPT1, CD3G, LEPROTL1, EEF1A1
27729	B_NAIVE	IGLL5, CD79A, TCL1A, MS4A1, FCRLA, CD79B, LINC00926, HLA-DQB1, FCER2, FAM129C
28078	CD4_NAIVE	IL7R, LEF1, PIK3IP1, TCF7, LINC00861, NOSIP, CAMK4, CCR7, NDFIP1, LEPROTL1
28213	CD8_NAIVE	CD8B, CD8A, NELL2, CCR7, LEF1, APBA2, CD3D, CARS, LEPROTL1, PIK3IP1
29149	T_REG	IL32, TNFRSF4, CCR10, DUSP4, ITGB1, FOXP3, CD3D, CORO1B, SIT1, AQP3
29287	IMMATURE MONO_CLAS	S100A12, S100A8, S100A9, RBP7, VCAN, PLBD1, CD14, SLC11A1, CYP1B1, MNDA
29666	CD4_NAIVE	IL7R, LEF1, LINC00861, TCF7, LRRN3, PIK3IP1, CAMK4, NDFIP1, NOSIP, MAL

Supplemental Table 1. Top 10 most highly enriched genes.

29821	CD4_NAIVE	LEF1, CD3G, TCF7, NOSIP, CD3D, CCR7, CD27, CD3E, LDLRAP1, LAT
30019	MONO_INT	LGALS2, CTSS, FGL2, SERPINA1, NEAT1, TYMP, CPVL, SAT1, CLEC7A, CD14
30460	NK	GNLY, FGFBP2, KLRF1, GZMH, PRF1, KLRD1, NKG7, GZMB, SPON2, FCGR3A
31097	PLASMA	TNFRSF17, GPRC5D, DERL3, IGF1, GLDC, HRASLS2, SPACA3, SDC1, MZB1, TXNDC5
31499	MONO_INT	LGALS2, CPVL, FGL2, SERPINA1, CTSS, NEAT1, TYMP, MARCO, CST3, SAT1
31568	CD8_CENTRAL_MEMORY	GZMK, DUSP2, IL32, LYAR, IL7R, CXCR3, CCL5, CD3D, CD3E, B2M
32206	CD4_TERM_EFFECT	NKG7, GZMH, GZMA, GNLY, CTSW, PRF1, CCL5, CST7, FGFBP2, KLRD1
34390	CD4_NAIVE	LEF1, TCF7, NOSIP, CD3G, CCR7, LDLRAP1, PIK3IP1, CD3E, CD3D, GIMAP7

Table S2. Reference marker genes used for reference cell annotation.

Annotation	Primary markers	Additional markers
HSC	*AVP* [1][a]	*CRHBP* [2][b]
MPP	*SPINK2* [3][b]	
CMP	*GATA2* [4][c]	
MEP	*CNRIP1* [5][d]	
Erythrocyte	*HBB* [6][d]	*AHSP*, *CA1* [6][d]
GMP	*CTSG* [7][d]	
Basophil	*HDC* [8][e]	*CLC* [8][e]
MPP	*RETN* [9][b]	
Immature monocyte	*S100A9* [10][b]	*S100A12, S100A8* [10][b]
Classical monocyte	*VCAN* [8][e]	*CD14* [8][e]
Intermediate monocyte	*SAT1* [11][b]	
Non-classical monocyte	*C1QA* [8][e]	
CLP (pro-B)	*AKAP12* [12][b]	
Pre-B	*VPREB1* [13][c]	
Transitional B	*DTX1* [14][b]	
Naive B	*TCL1A* [8][e]	*FCER2* [8][e]
Memory B	*MS4A1* [8][e]	Absence of *TCL1A*
Plasma	*TNFRSF17* [15][e]	*GPRC5D* [16][g]
Naive CD4+	*LEF1* [8][f]	
CD4+	*LTB* [8][e]	Absence of *TNFRSF4* [8][e]
Th17	*TNFRSF4* [8][e]	
CD4+ TE	*GZMH* [8][e]	Express *CD4* [8][e]
Treg	*DUSP4* [8][e]	
Naive CD8	*NELL2* [8][e]	
CD8+ MAIT	*SLC4A10* [17][d]	
CD8+CM	*CCL5* [8][e]	Express *IL-32* and *IL7R* [8][e]
CD8+ EM	*DUSP2* [8][e]	
CD8+ TE	*NKG7* [8][e]	*CD8* [8][e]
NK	*GNLY* [8][e]	
pDC	*LILRA4* [8][e]	
mDC	*ENHO* [8][e]	

[a]Differentially expressed in scRNA-seq clusters.
[b]Bulk RNA quantification of FACS-sorted cells.
[c]Literature review.
[d]Differentially expressed in scRNA-seq of FACS-sorted cells.
[e]The Monaco scaled dataset from the Human Blood Atlas.
[f]mRNA-abaundance measured in human cell lines.
[g]Analysis of the Genotype Tissue Expression (GTEx) data base.

References

[1] Zheng, S., Papalexi, E., Butler, A., Stephenson, W. & Satija, R. Moleculartransitions in early progenitors during human cord blood hematopoiesis. *Molecular Systems Biology* **14**, e8041 (2018).

[2] Toren, A. G. et al. CD133-positive hematopoietic stem cell ”stemness” genes contain many genes mutated or abnormally expressed in leukemia. *Stem cells* **23**(8), 1142–53 (2005).

[3] He, X. et al. Differential gene expression profiling of CD34+ CD133+ umbilical cord blood hematopoietic stem progenitor cells. *Stem cells and development* **14**(2), 188–98 (2005).

[4] Suzuki, M., Shimizu, R. & Yamamoto, M. Transcriptional regulation by GATA-1 and GATA-2 during erythropoiesis. *International Journal of Hematology* **93**, 150–155 (2011).

[5] Lu, Y.-C.et al. The molecular signature of megakaryocyte-erythroid progenitors reveals a role for the cell cycle in fate specification. *Cell reports* **25**, 2083 – 2093.e4 (2018).
[6] Velten, L. et al. Human haematopoietic stem cell lineage commitment is acontinuous process. *Nature cell biology* **19**, 271 – 281 (2017).
[7] Pellin, D. et al. A comprehensive single cell transcriptional landscape of human hematopoietic progenitors. *Nature Communications* **10** (2019).
[8] Uhlén, M. et al. A genome-wide transcriptomic analysis of protein-coding genes in human blood cells. *Science* **366**(6472), eaax9198 (2019).
[9] Mello, F. V. et al. Maturation-associated gene expression profiles along normal human bone marrow monopoiesis. *British Journal of Haematology* **176**, 464–474 (2017).
[10] Zhao, F. et al. S100a9 a new marker for monocytic human myeloid derived supressor cells. *Immunology* **136**, 176–183 (2012).
[11] Zawada, A. M. et al. Supersage evidence for CD14++CD16+ monocytes as a third monocyte subset. *Blood* **118(**12), e50–61 (2011).
[12] Månsson, R. et al. Positive intergenic feedback circuitry, involving EBF1 and FOXO1, orchestrates B-cell fate. *Proceedings of the National Academy of Sciences* **109**, 21028 – 21033 (2012).
[13] Clark, M. R., Mandal, M., Ochiai, K. & Singh, H. Orchestrating b celllymphopoiesis through interplay of il-7 receptor and pre-b cell receptorsignalling.Nature reviews. Immunology14, 69—80 (2014).
[14] Suryani, S. et al. Differential expression of CD21 identifies developmentally and functionally distinct subsets of human transitional B cells. *Blood* **1153**, 519–29 (2010).
[15] Laabi, Y. et al. The BCMA gene, preferentially expressed during b lym-phoid maturation, is bidirectionally transcribed. *Nucleic Acids Research* **22**, 1147–1154 (1994).
[16] Smith, E. L. et al. Gprc5d is a target for the immunotherapy of multiple myeloma with rationally designed CAR T cells. *Science Translational Medicine* **11,** (2019).
[17] Huang, H. et al. Select sequencing of clonally expanded CD8+ t cells revealslimits to clonal expansion. *Proceedings of the National Academy of Sciences of the United States of America* **116**, 8995–9001 (2019).

Table S3. Annotations and evidence for the marker gene status for genes that are ranked in the top 25 enriched genes in at least one reference cell by SEMITONES.

Gene	Annotation	Rank	Evidence?	Reference
A2M-AS1	NK and memory T cells	21	Yes	[1]
AC002454.1	MPP, CMP, CLP	2	Yes	[2]
AC084018.1	CD4 naive, CD8 naive	25	No	
AC104699.1	Plasma cell	13	Yes	[2]
AEBP1	Transitional B	20	No	
AMPD1	Plasma cell	22	Yes	[3]
ANGPT1	HSC, MPP	18	Yes	[4]
ANK1	Erythrocyte	17	Yes	[5]
ANKRD28	HSC, MPP, CMP	5	Yes	[4]
ANKRD55	CD4 naive	17	Yes	[3]
AP3M2	CD4 memory	4	Yes	[3]
APBB2	Transitional B	6	No	
APOC1	MEP, Erythrocyte	2	Yes	[6]
AURKB	Cycling cells	7	No	[a]
AZU1	GP (neutrophil)	4	Yes	[3]
BIRC5	Cycling cells	4	No	[a]
BMP3	Transitional B	3	Yes	[7] [b]
C1QA	Non-classical monocytes	3	Yes	[3]
C1QB	Intermediate and non-classical monocyte	16	Yes	[3]
C1QTNF4	HSC, MPP, GMP	2	Yes	[2,4,8][c]
C20orf27	GP, Monocyte, mDC	17	Yes	[3]
C7orf57	pre B	21	No	
CARS	CD8 naive	8	No	
CCNA2	Cycling cells	22	No	[a]
CCNB2	Cycling cells	13	No	[a]
CDC20	Cycling cells	10	No	[a]

Table S3. continued

Gene	Annotation	Rank	Evidence?	Reference
CDH7	MPP	23	Yes	[9][d,e]
CDK6	HSC, MPP, CMP, CLP	4	Yes	[4]
CENPA	Cycling cells	11	No	[a]
CENPF	Cycling cells	2	No	[a]
CKAP2L	Cycling cells	10	No	[a]
CKS1B	Cycling cells	3	No	[a]
CLC	Eosinophil/Basophil/Mast cell-lineage	1	Yes	[3]
CLDN3	Cycling cells	18	No	[a]
CLEC11A	Monocyte progenitor	9	Yes	[3]
CLEC14A	Pro B	15	No	
COL23A1	GP (neutrophil)	7	No	
CRHBP	HSC/MPP	2	Yes	[10]
CRIP2	CD4 memory, B memory	7	Yes	[3]
CRYGD	HSC, MPP, CLP	11	Yes	[11]
CTSL	Non-classical monocytes	18	Yes	[3]
CTSV	pDC (progenitors)	3	Yes	[3]
CYGB	CLP, pro B	2	Yes	[2]
CYTL1	HSC, MPP, CMP	4	No	
DACT1	CD4 naive, CD4 memory	12	Yes	[3]
DTX1	Transitional B	1	Yes	[12]
DUSP26	Pro B	12	No	
E2F1	Pre B	18	Yes	[13]
E2F2	Cycling cells	15	No	[a]
EEF1A1	CD8 naive, CD4 naive/CD4 memory	6	Yes	[3]
EMID1	CMP	15	Yes	[14]
ENHO	mDC	2	Yes	[3]
EPCAM	Erythrocyte	9	Yes	[15]

Table S3. continued

Gene	Annotation	Rank	Evidence?	Reference
EXD3	Eosinophil, Basophil, Mast cell-lineage	14	No	
FAM178B	Erythrocyte	17	Yes	[2]
FAM83F	Eosinophil/Basophil/Mast cell-lineage	11	Yes	[3]
FAM92B	Plasma cell	12	Yes	[15]
FES	MP/Classical monocytes	15	Yes	[3]
FHL2	MPP, MEP, Erythrocyte	5	No	
FMNL2	Intermediate and non-classical monocyte	21	Yes	[3]
FOLR3	MP	6	Yes	[3]
FXYD2	NK/CD8 memory	18	Yes	[3]
GATA1	CMP, MEP	5	Yes	[16]
GNG11	MEP	22	Yes	[17]
HBA2	Erythrocyte	20	Yes	[5]
HBD	Erythrocyte	1	Yes	[5]
HBQ1	Erythrocyte	4	Yes	[5]
HEMGN	MPP	25	Yes	[18]
HES4	Non-classical monocytes	2	Yes	[3]
HIST1H2AC	Transitional B	18	No	
HLA-DQA2	mDC	14	Yes	
HLF	HSC	15	Yes	[19]
HMHB1	Pro B	7	No	
HMMR	Cycling cells	5	No	[a]
HOXA9	MPP	18	Yes	[20]
HRASLS2	Plasma cell	6	Yes	[21]
HTR1F	HSC, MPP	10	Yes	[4]
IGFBP2	CMP	11	Yes	[22]
IGSF10	HSC, MPP, MEP	23	Yes	[2]
KCNH2	Erythrocyte	10	Yes	[2]

Table S3. continued

Gene	Annotation	Rank	Evidence?	Reference
KCTD12	mDC, Classical and intermediate monocyte	13	Yes	[3]
KIAA0087	CLP	14	Yes	[2]
KIAA0930	Moncoyte, mDC	21	Yes	[3]
KIF15	Cycling cells	25	No	[a]
KLF1	MEP, Erythrocyte	4	Yes	[23]
KREMEN2	Transitional B	25	No	
LINC00114	Pro B	9	No	
LINC00582	Plasma cell	14	Yes	[2]
LINC00861	CD4 naive, CD8 naive	3	No	
LINC00926	B cell	1	Yes	[2,24,25]
LINC01013	Pro B	8	Yes	[26]
LINC01133	Erythrocyte	15	Yes	[27]
LMO4	Eosinophil/Basophil/Mast cell-lineage	6	Yes	[28]
LTBP1	Eosinophil/Basophil/Mast cell-lineage	21	Yes	[3]
LYPD2	Non-classical monocytes	8	Yes	[3]
MALAT1	Lymphocyte-enriched	22	No	
MDK	HSC, MPP	4	Yes	[11]
MEG3	HSC, MPP	3	Yes	[29]
MICALL2	GP (neutrophil)	21	No	
MIR181A1HG	MPP, CLP, proB, preB	24	No	
MMP2	HSC, CMP	19	No	
MSI2	HSC, MPP, CLP	17	Yes	[4]
MSRB3	HSC, MPP, CMP	11	Yes	[4]
MYCT1	MPP/CMP	8	Yes	[30]
MYL4	Transitional B	21	No	
MYLK	Pro B	16	No	
MYO1C	Transitional B	17	No	

Table S3. continued

Gene	Annotation	Rank	Evidence?	Reference
NEAT1	Monocyte	4	Yes	[25]
NECAB1	MEP, Erythrocyte	6	No	
NME1	CMP, MEP, CLP	17	No	
NMU	Erythrocyte	7	Yes	[31]
NPM3	HSC, MPP, CMP, CLP	25	No	
NPR3	HSC, MPP	8	Yes	[4]
NPW	GMP/GP	9	No	
NREP	MPP, CMP, CLP	13	Yes	[32]
NUSAP1	Cycling cells	3	No	[a]
OR10J3	Eosinophil/Basophil/Mast cell-lineage	12	Yes	[3]
P4HA2	Pro B, Pre B	19	No	
PBX1	MPP	9	Yes	[33][d]
PDZD8	MPP, MEP, GMP	14	No	
PKIB	mDC	4	Yes	[3]
PKLR	Erythrocyte	24	Yes	[5]
PLK1	Cycling cells	14	No	[a]
PP1R17	Non-classical monocyte	19	Yes	[3]
PRKAR2B	MEP, Erythrocyte	6	Yes	[34]
PROC	pDC	18	Yes	[3]
PRRT4	GP (neutrophil)	15	No	
PRSS1	HSC, MPP	1	Yes	[3]
PRSS3	HSC	3	No	
PRSS57	GP (neutrophil)	3	Yes	[35]
RAB13	HSC, MPP	17	Yes	[36]
RAB32	GMP, Monocyte, mDC, Eosinophil/Basophil/Mast cell-lineage	14	Yes	[3]
RAG1	Pro B, Pre B	13	Yes	[37]
RAG2	Pre B	24	Yes	[38]

Table S3. continued

Gene	Annotation	Rank	Evidence?	Reference
RAMP1	MPP, CMP, CLP	25	Yes	[39]
RBPMS2	MEP, Erythrocyte	12	No	
REG4	CD8 naive	4	Yes	[3]
RHAG	Erythrocyte	5	Yes	[40]
RRAS	Non-classical monocyte	17	Yes	[3]
RRM2	Cycling cells	5	No	[a]
RTN1	Monocyte, mDC	18	Yes	[3]
SCGB3A1	Naive CD8, Memory CD4, Non-classical monocyte	22	Yes	[3]
SCNN1B	Plasma cell (plasmablast)	20	Yes	[3]
SCT	pDC	1	Yes	[3]
SERPINB10	GMP	19	Yes	[41]
SFRP5	Naive B	14	Yes	[3]
SH2D4B	Pro B	14	No	
SHD	pDC	15	Yes	[3]
SLC8A1-AS1	Pro B	17	No	
SLPI	GP (neutrophil)	25	Yes	[3]
SMIM1	MEP, Erythrocyte	23	Yes	[42]
SMIM10	MEP, Erythrocyte	13	Yes	[2]
SNHG8	HSC, MPP	18	Yes	[17]
SPACA3	Plasma cell (plasmablast)	7	Yes	[3]
SPAG4	Cycling cells	23	No	[a]
SPC25	Cycling cells	19	No	[a]
SPINK2	HSC, MPP, CMP, CLP	1	Yes	[4]
STAB1	Classical and intermediate monocyte	11	Yes	[3]
TCL1B	Transitional B/Naive B	9	Yes	[3,43][d]
TFPI	MPP	22	Yes	[44]
TFR2	MEP, Erythrocyte	18	Yes	[45]

Table S3. continued

Gene	Annotation	Rank	Evidence?	Reference
TK1	Cycling cells	15	No	[a]
TM4SF1	MPP	18	No	
TMEM14C	MEP, Erythrocyte	11	Yes	[46]
TMEM56	MEP, Erythrocyte	12	Yes	[2,15]
TMSB15A	Pre B	24	No	
TOP2A	Cycling cells	2	No	[a]
TPM1	MEP, Erythrocyte	12	Yes	[47]
TPX2	Cycling cells	12	No	[a]
TTLL7	Eosinophil/Basophil/Mast cell-lineage	22	Yes	[3]
UBE2C	Cycling cells	1	No	[a]
UMODL1	CLP	6	No	
UROD	MEP, Erythrocyte	20	Yes	[5]
WASF1	Transitional B	24	No	
ZFAS1	HSC, MPP, CMP, GMP	6	No	
ZNF703	Non-classical monocyte	24	Yes	[3]
ZNFD385D	MEP	14	Yes	[2][f]

[a] Cell cycle genes are never considered marker genes
[b] Small pre-B cells
[c] Non-cyling HSC
[d] Mouse
[e] Zebrafish
[f] CMP and platelets

References

[1] Grennan, A. K. Genevestigator. Facilitating Web-Based Gene-Expression Analysis. *Plant Physiology* 141, 1164–1166 (2006).

[2] Sommarin, M. N. *et al.* Single-cell multiomics reveals distinct cell states at the top of human hematopoietic hierarchy. *bioRxiv* (2021).

[3] Uhlén, M. *et al.* A genome-wide transcriptomic analysis of protein-coding genes in human blood cells. *Science* 366 (2019).

[4] Huang, T.-S. *et al.* Functional network reconstruction reveals somatic stemness genetic maps and dedifferentiation-like transcriptome reprogramming induced by gata2. *STEM CELLS* 26, 1186–1201 (2008).

[5] Bethesda (MD): National Library of Medicine (US), N. C. f. B. I. Gene [internet] (2004). URL https://www.ncbi.nlm.nih.gov/gene/. Accessed on 8 April 2021.

[6] Lai, S. *et al.* Comparative transcriptomic analysis of hematopoietic system between human and mouse by microwell-seq. *Cell Discovery* 4 (2018).

[7] Stelzer, G. *et al.* The genecards suite: From gene data mining to disease genome sequence analyses. *Current Protocols in Bioinformatics* 54, 1.30.1– 1.30.33 (2016).

[8] Venkatasubramanian, M., Chetal, K., Schnell, D. J., Atluri, G. & Salomonis, N. Resolving single-cell heterogeneity from hundreds of thousands of cells through sequential hybrid clustering and NMF. *Bioinformatics* 36, 3773–3780 (2020).

[9] Hsu, J. *et al.* Chd7 and runx1 interaction provides a braking mechanism for hematopoietic differentiation. *Proceedings of the National Academy of Sciences* 117, 23626–23635 (2020).

[10] Pellin, D. *et al.* A comprehensive single cell transcriptional landscape of human hematopoietic progenitors. *Nature Communications* 10 (2019).

[11] Toren, A. *et al.* Cd133-positive hematopoietic stem cell "stemness" genes contain many genes mutated or abnormally expressed in leukemia. *STEM CELLS* 23 (2005).

[12] Suryani, S. *et al.* Differential expression of CD21 identifies developmentally and functionally distinct subsets of human transitional B cells. *Blood* 115, 519–529 (2010).

[13] Hystad, M. *et al.* Characterization of early stages of human b cell development by gene expression profiling. *The Journal of Immunology* 182, 5882 – 5882 (2009).

[14] Danis, E. *et al.* Ezh2 controls an early hematopoietic program and growth and survival signaling in early t cell precursor acute lymphoblastic leukemia. *Cell reports* 14, 1953 – 1965 (2016).

[15] Franzén, O., Gan, L.-M. & Bjo¨rkegren, J. L. M. PanglaoDB: a web server for exploration of mouse and human single-cell RNA sequencing data. *Database* 2019 (2019). Baz046.

[16] Akashi, K., Traver, D., Miyamoto, T. & Weissman, I. A clonogenic common myeloid progenitor that gives rise to all myeloid lineages. *Nature* 404, 193– 197 (2000).

[17] Zhao, Y. *et al.* Single-cell transcriptomic landscape of nucleated cells in umbilical cord blood. *GigaScience* 8 (2019).

[18] Cabezas-Wallscheid, N. *et al.* Identification of regulatory networks in hscs and their immediate progeny via integrated proteome, transcriptome, and dna methylome analysis. *Cell stem cell* 15 4, 507–522 (2014).

[19] Yokomizo, T. *et al.* Hlf marks the developmental pathway for hematopoietic stem cells but not for erythro-myeloid progenitors. *The Journal of Experimental Medicine* 216, 1599 – 1614 (2019).

[20] Ramos-Mejia, V. *et al.* Hoxa9 promotes hematopoietic commitment of human embryonic stem cells. *Blood* 124 20, 3065–75 (2014).

[21] Covens, K. *et al.* Characterization of proposed human b-1 cells reveals pre-plasmablast phenotype. *Blood* 121, 5176–5183 (2013).

[22] Huynh, H. *et al.* IGF binding protein 2 supports the survival and cycling of hematopoietic stem cells. *Blood* 118, 3236–3243 (2011).

[23] Magor, G. W. *et al.* Klf1-null neonates display hydrops fetalis and a deranged erythroid transcriptome. *Blood* 125 15, 2405–17 (2015).

[24] Nirmal, A. J. *et al.* Immune cell gene signatures for profiling the microenvironment of solid tumors. *Cancer Immunology Research* 6, 1388 – 1400
(2018).

[25] Zhang, M. J., Ntranos, V. & Tse, D. N. Determining sequencing depth in a single-cell rna-seq experiment. *Nature Communications* 11 (2020).

[26] Petri, A. *et al.* Long noncoding rna expression during human B-cell development. *PLOS ONE* 10, 1–19 (2015).

[27] Ren, Y. *et al.* The dynamic interactive network of long non-coding rnas and chromatin accessibility facilitates erythroid differentiation. *bioRxiv* (2021).

[28] Paul, F. *et al.* Transcriptional heterogeneity and lineage commitment in myeloid progenitors. *Cell* 163, 1663–1677 (2015).

[29] Ali, M. A. E. *et al.* Functional dissection of hematopoietic stem cell populations with a stemness-monitoring system based on ns-gfp transgene expression. *Scientific Reports* 7 (2017).

[30] Gambone, J. E., Dusaban, S. S., Loperena, R., Nakata, Y. & Shetzline, S. E. The c-Myb target gene neuromedin U functions as a novel cofactor during the early stages of erythropoiesis. *Blood* 117, 5733–5743 (2011).

[31] Korniotis, S. *et al.* Mobilized multipotent hematopoietic progenitors stabilize and expand regulatory t cells to protect against autoimmune encephalomyelitis. *Frontiers in Immunology* 11 (2020).

[32] Ficara, F. *et al.* Pbx1 restrains myeloid maturation while preserving lymphoid potential in hematopoietic progenitors. *Journal of Cell Science* 126, 3181–3191 (2013).

[33] Tyser, R. C. *et al.* A spatially resolved single cell atlas of human gastrulation. *bioRxiv* (2020).

[34] Perera, N. C. *et al.* Nsp4 is stored in azurophil granules and released by activated neutrophils as active endoprotease with restricted specificity. *The Journal of Immunology* 191, 2700–2707 (2013).

[35] Forsberg, E. C. *et al.* Differential expression of novel potential regulators in hematopoietic stem cells. *PLOS Genetics* 1, e28 (2005).

[36] Johnson, K. *et al.* Il-7 functionally segregates the pro-b cell stage by regulating transcription of recombination mediators across cell cycle. *The Journal of Immunology* 188, 6084–6092 (2012).

[37] Grawunder, U. *et al.* Down-regulation of rag1 and rag2 gene expression in preb cells after functional immunoglobulin heavy chain rearrangement. *Immunity* 3, 601–608 (1995).

[38] Harzenetter, M. D. *et al.* Regulation and function of the cgrp receptor complex in human granulopoiesis. *Experimental Hematology* 30, 306–312 (2002).

[39] Avent, N. D. & Reid, M. E. The Rh blood group system: a review. *Blood* 95, 375–387 (2000).

[40] Cytlak, U. *et al.* Differential irf8 transcription factor requirement defines two pathways of dendritic cell development in humans. *Immunity* 53, 353 – 370.e8 (2020).

[41] Nylander, A., Leznicki, P., Vidovic, K., High, S. & Olsson, M. Smim1, carrier of the vel blood group, is a tail-anchored transmembrane protein and readily forms homodimers in a cell-free system. *Bioscience Reports* 40 (2020).

[42] Kang, S.-M. *et al.* Impaired t- and b-cell development in tcl1-deficient mice. *Blood* 105, 1288–1294 (2005).

[43] Georgantas, R. W. *et al.* Microarray and serial analysis of gene expression analyses identify known and novel transcripts overexpressed in hematopoietic stem cells. *Cancer Research* 64, 4434–4441 (2004).

[44] Richard, C. & Verdier, F. Transferrin receptors in erythropoiesis. *International Journal of Molecular Sciences* 21 (2020).

[45] Yien, Y. Y. *et al.* Tmem14c is required for erythroid mitochondrial heme metabolism. *The Journal of clinical investigation* 12410, 4294–304 (2014).

[46] Thom, C. *et al.* Tropomyosin 1 Genetically Constrains in Vitro Megakaryopoiesis. *Blood* 134, 3612–3612 (2019).

[47] Sui, Z. *et al.* Stabilization of F-actin by tropomyosin isoforms regulates the morphology and mechanical behavior of red blood cells. *Molecular Biology of the Cell* 28, 2531-2542 (2017).
